# Simplified HIV Testing and Treatment in China: Analysis of Mortality Rates Before and After a Structural Intervention

**DOI:** 10.1371/journal.pmed.1001874

**Published:** 2015-09-08

**Authors:** Zunyou Wu, Yan Zhao, Xianmin Ge, Yurong Mao, Zhenzhu Tang, Cynthia X. Shi, Chi Chen, Yong Li, Xuejun Qiu, Guide Nong, Shanhui Huang, Shen Luo, Shaohui Wu, Wenzhen He, Mingjie Zhang, Zhiyong Shen, Xia Jin, Jian Li, Ron Brookmeyer, Roger Detels, Julio Montaner, Yu Wang

**Affiliations:** 1 National Center for AIDS/STD Control and Prevention, Chinese Center for Disease Control and Prevention, Beijing, China; 2 Guangxi Bureau of HIV/AIDS, Guangxi Health Department, Nanning, China; 3 Guangxi Center for Disease Control and Prevention, Nanning, China; 4 Guangxi Antiretroviral Treatment Center, Liuzhou, China; 5 Zhongshan County Health Bureau, Zhongshan, China; 6 Zhongshan County Center for Disease Control and Prevention, Zhongshan, China; 7 Zhongshan County General Hospital, Zhongshan, China; 8 Pubei County Health Bureau, Pubei, China; 9 Pubei County Center for Disease Control and Prevention, Pubei, China; 10 Pubei County General Hospital, Pubei, China; 11 Department of Biostatistics, Fielding School of Public Health, University of California at Los Angeles, Los Angeles, California, United States of America; 12 Department of Epidemiology, Fielding School of Public Health, University of California at Los Angeles, Los Angeles, California, United States of America; 13 BC Centre for Excellence in HIV/AIDS, University of British Columbia, Vancouver, British Columbia, Canada; 14 Chinese Center for Disease Control and Prevention, Beijing, China; Boston University, UNITED STATES

## Abstract

**Background:**

Multistage stepwise HIV testing and treatment initiation procedures can result in lost opportunities to provide timely antiretroviral therapy (ART). Incomplete patient engagement along the continuum of HIV care translates into high levels of preventable mortality. We aimed to evaluate the ability of a simplified test and treat structural intervention to reduce mortality.

**Methods and Findings:**

In the “pre-intervention 2010” (from January 2010 to December 2010) and “pre-intervention 2011” (from January 2011 to December 2011) phases, patients who screened HIV-positive at health care facilities in Zhongshan and Pubei counties in Guangxi, China, followed the standard-of-care process. In the “post-intervention 2012” (from July 2012 to June 2013) and “post-intervention 2013” (from July 2013 to June 2014) phases, patients who screened HIV-positive at the same facilities were offered a simplified test and treat intervention, i.e., concurrent HIV confirmatory and CD4 testing and immediate initiation of ART, irrespective of CD4 count. Participants were followed for 6–18 mo until the end of their study phase period. Mortality rates in the pre-intervention and post-intervention phases were compared for all HIV cases and for treatment-eligible HIV cases. A total of 1,034 HIV-positive participants (281 and 339 in the two pre-intervention phases respectively, and 215 and 199 in the two post-intervention phases respectively) were enrolled. Following the structural intervention, receipt of baseline CD4 testing within 30 d of HIV confirmation increased from 67%/61% (pre-intervention 2010/pre-intervention 2011) to 98%/97% (post-intervention 2012/post-intervention 2013) (all *p* < 0.001 [i.e., for all comparisons between a pre- and post-intervention phase]), and the time from HIV confirmation to ART initiation decreased from 53 d (interquartile range [IQR] 27–141)/43 d (IQR 15–113) to 5 d (IQR 2–12)/5 d (IQR 2–13) (all *p* < 0.001). Initiation of ART increased from 27%/49% to 91%/89% among all cases (all *p* < 0.001) and from 39%/62% to 94%/90% among individuals with CD4 count ≤ 350 cells/mm^3^ or AIDS (all *p* < 0.001). Mortality decreased from 27%/27% to 10%/10% for all cases (all *p* < 0.001) and from 40%/35% to 13%/13% for cases with CD4 count ≤ 350 cells/mm^3^ or AIDS (all *p* < 0.001). The simplified test and treat intervention was significantly associated with decreased mortality rates compared to pre-intervention 2011 (adjusted hazard ratio [aHR] 0.385 [95% CI 0.239–0.620] and 0.380 [95% CI 0.233–0.618] for the two post-intervention phases, respectively, for all newly diagnosed HIV cases [both *p* < 0.001], and aHR 0.369 [95% CI 0.226–0.603] and 0.361 [95% CI 0.221–0.590] for newly diagnosed treatment-eligible HIV cases [both *p* < 0.001]). The unit cost of an additional patient receiving ART attributable to the intervention was US$83.80. The unit cost of a death prevented because of the intervention was US$234.52.

**Conclusions:**

Our results demonstrate that the simplified HIV test and treat intervention promoted successful engagement in care and was associated with a 62% reduction in mortality. Our findings support the implementation of integrated HIV testing and immediate access to ART irrespective of CD4 count, in order to optimize the impact of ART.

## Abstract 2

In a before and after analysis, Zunyou Wu and colleagues assess the impact on mortality of an HIV test and treat intervention in two counties in Guangxi, China.

## Editors' Summary

**Background:**

Every year, about 2.1 million people (mostly living in resource-limited countries) are newly infected with HIV, the virus that causes AIDS and that has killed 39 million people over the past three decades. HIV, which is usually transmitted through unprotected sex with an infected individual, gradually destroys CD4 lymphocytes and other immune system cells, leaving HIV-positive individuals susceptible to other infections. Early in the AIDS epidemic, most HIV-positive individuals died within ten years of infection. Then, in 1996, effective antiretroviral therapy (ART) became available, and, for people living in high-income countries, HIV became a chronic condition. But ART was expensive, so HIV/AIDS remained largely untreated and fatal in resource-limited countries. In 2003, the international community began to work towards achieving universal ART coverage. By 2013, about 12.9 million people living with HIV (a third of all HIV-positive people) had access to ART, and the rate of AIDS-related deaths had fallen by a third from its 2005 peak.

**Why Was This Study Done?:**

Unfortunately, in many countries, late diagnosis of HIV infection, incomplete linkage to care after diagnosis, and high rates of loss to follow-up before and after ART initiation remain major barriers to effective HIV/AIDS treatment and to maximization of the preventative benefits of ART: as well as keeping HIV-positive people healthy, ART also reduces their chances of transmitting HIV to a sexual partner. Here, the researchers investigate whether a simplified “test and treat” intervention can reduce HIV/AIDS mortality (death) rates in China by reducing these barriers. Currently, CD4 testing is offered to people in China only after an initial HIV diagnosis has been confirmed using a second type of test. This standard-of-care policy introduces a delay into ART initiation because a CD4 count below 350 cells/μl blood is the primary determinant of eligibility for treatment through the Chinese national free ART program. By contrast, the simplified test and treat intervention, which is designed to be completed within a week of the patient’s first positive HIV test result, incorporates immediate HIV confirmatory testing, pre-ART CD4 testing, pre-treatment counseling, and ART initiation regardless of CD4 count.

**What Did the Researchers Do and Find?:**

The researchers followed about 1,000 patients who tested positive for HIV at health care facilities in two counties in Guangxi (one of the Chinese provinces most heavily affected by HIV/AIDS) in two 12-month pre-intervention phases, during which patients followed the standard-of-care process, and two 12-month post-intervention phases, during which patients were offered the simplified test and treat intervention. About 65% and 97% of the patients received baseline CD4 testing during the pre-intervention and post-intervention phases, respectively. Following the structural intervention, the time from HIV confirmation to ART initiation decreased from around 50 days to five days, and the proportion of individuals who initiated ART increased from below 36% to above 90% among all the patients and from below 47% to above 93% among patients eligible for treatment under the standard-of-care policy. Notably, the mortality rate decreased from about 26% to about 10% among all the study participants following the intervention, and from about 37% to about 13% among the participants eligible for ART under the standard-of-care policy. Finally, the researchers estimated that the cost of each death prevented by the intervention was about US$234.52 over the study period; importantly, most of this cost was accrued during the initial year of the intervention.

**What Do These Findings Mean?:**

These findings indicate that, in the two Chinese counties involved in this study, the simplified test and treat intervention—which incorporated a streamlined, standardized time frame for HIV diagnosis and expanded access to ART—promoted successful engagement in care among HIV-positive individuals and was associated with a 62% reduction in mortality. Moreover, the intervention required very little further investment once it had been set up and should, therefore, be sustainable. Because the design of the simplified test and treat intervention took into account the characteristics of the HIV epidemic and the health care structure in China, these findings may not be fully generalizable to other countries. In addition, reliance on a pre-intervention/post-intervention study design, rather than a controlled trial, may limit the accuracy of these findings. Nevertheless, these results suggest that the implementation of integrated HIV testing and immediate access to ART regardless of CD4 count has the potential to optimize the individual and public health impacts of ART by ensuring that fewer patients are lost along the multistage continuum of HIV testing and treatment.

**Additional Information:**

This list of resources contains links that can be accessed when viewing the PDF on a device or via the online version of the article at http://dx.doi.org/10.1371/journal.pmed.1001874.

- Information is available from the US National Institute of Allergy and Infectious Diseases on HIV infection and AIDS
- NAM/aidsmap provides basic information about HIV/AIDS, summaries of recent research findings on HIV care and treatment, and personal stories about living with HIV/AIDS
- Information is available from Avert, an international AIDS charity, on many aspects of HIV/AIDS, including information on universal access to ART and on HIV/AIDS in China; Avert also provides personal stories about living with HIV/AIDS
- The World Health Organization provides information on all aspects of HIV/AIDS (in several languages), including its Consolidated Guidelines on the Use of Antiretroviral Therapy for Treating and Preventing HIV Infection, its recently released consolidated guidelines on HIV testing, and information on the WHO/UNAIDS Treatment 2.0 strategy, an initiative to expand access to HIV testing and ART
- The UNAIDS Fast-Track Strategy to End the AIDS Epidemic by 2030 provides up-to-date information about the AIDS epidemic and efforts to halt it, including progress towards universal access to antiretroviral therapy

## Introduction

In June 2010, the World Health Organization (WHO) and the Joint United Nations Programme on HIV/AIDS (UNAIDS) launched the Treatment 2.0 strategy, an initiative to expand access to HIV testing and antiretroviral therapy (ART) and to maximize the individual and public health benefits of modern HIV treatment [1]. Global experience over the past decade has confirmed the lifesaving benefits of ART for treating HIV-positive patients. However, late diagnosis, incomplete linkage to care, and loss to follow-up (LTFU) remain major clinical and public health challenges [2–4]. High rates of LTFU, before and after ART initiation, are relatively common in both high- and low-resource settings [5–9]. Moreover, late initiation of ART and high LTFU rates are significant drivers of mortality [10–13]. Despite nationwide scale-up of HIV programs in China over the last decade, the proportion of ART-eligible HIV-positive patients who receive treatment remains low. In 2009, it was estimated that 53.3% of HIV-positive individuals received a baseline CD4 count test within 6 mo of diagnosis [14], and among ART-eligible patients, treatment coverage was 63.4%, and the mortality rate was 14.2 per 100 person-years [12].

In China, as in many settings worldwide, patients are lost at each step along the continuum of HIV testing and care. This includes patients lost after not meeting ART eligibility criteria at the time of diagnosis. Other patients may meet treatment criteria but fail to initiate ART. Complicated HIV testing policies may be contributing to early LTFU. According to the current Chinese standard-of-care policies [15], CD4 testing is offered only after the HIV diagnosis has been confirmed through Western blot (WB) testing. This results in a structural delay to initiating ART because CD4 count is the primary measure used to determine eligibility for the Chinese National Free Antiretroviral Treatment Program (NFATP).

Although published studies have described the HIV care continuum, most studies have been either observational or focused on an intervention targeting a limited section of the HIV care cascade, such as increasing the proportion of participants receiving CD4 testing or the proportion initiating ART [16,17]. Strategic interventions to streamline HIV testing and treatment procedures should be designed to decrease LTFU and mortality. We designed a structurally simplified test and treat intervention, to be completed within a week of the first positive HIV screening test result, incorporating immediate HIV confirmatory testing, pre-ART CD4 testing, pretreatment counseling, and ART initiation regardless of CD4 count. The aim of this pilot study was to evaluate the effectiveness of the simplified test and treat intervention in reducing delays to treatment and decreasing mortality.

## Methods

### Study Design

**Fig 1:**
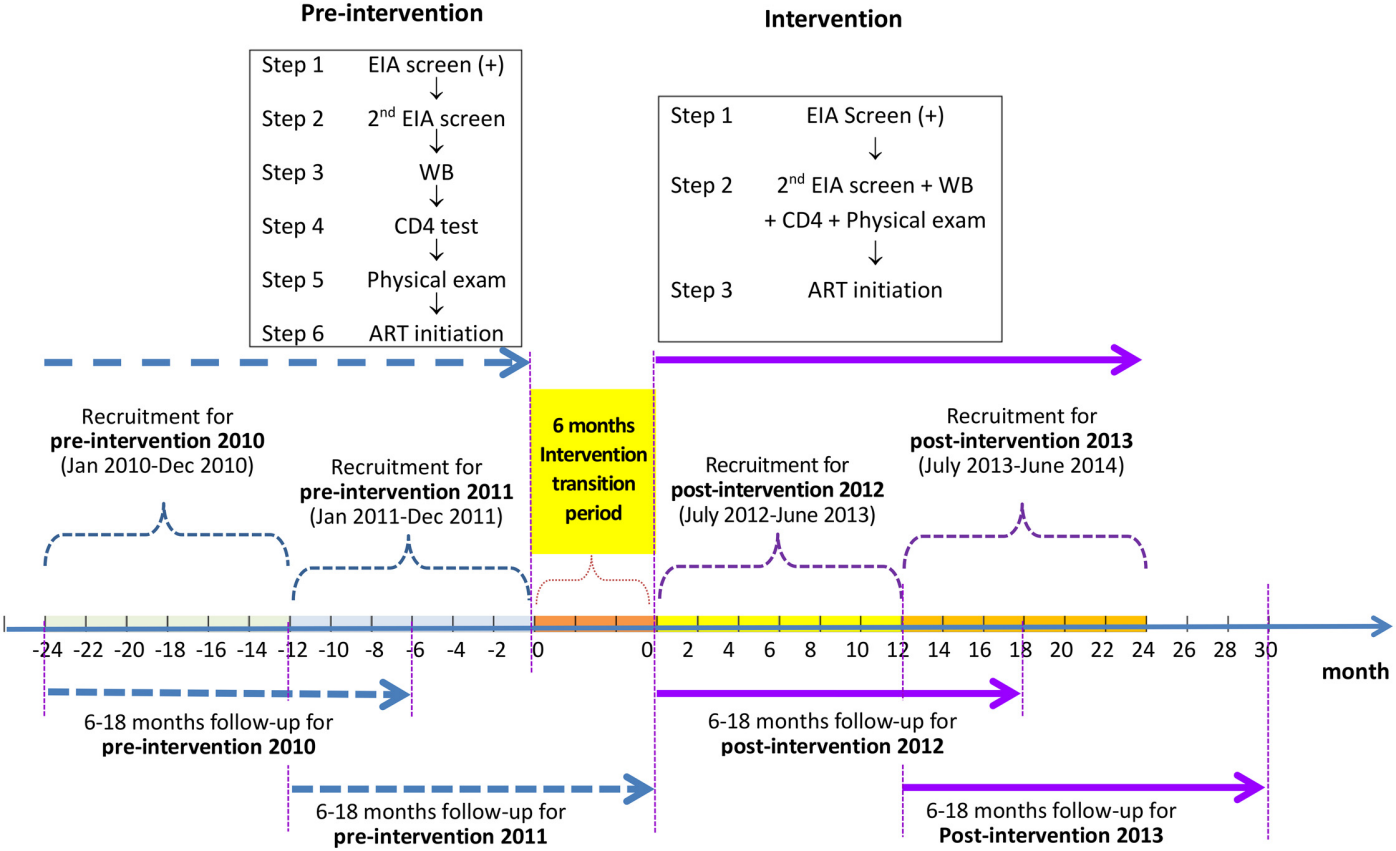
Study design of the simplified HIV test and treat intervention. Pre-intervention consisted of the standard of care; there were six steps from the enzyme immunoassay (EIA) screen to ART initiation, and the eligibility for ART was at CD4 count ≤ 350 cells/mm^3^. The simplified test and treat intervention comprised three steps from the enzyme immunoassay screen to ART initiation, regardless of CD4 level. In all four phases, participants were followed for a period of 6 to 18 mo, from the date of their WB confirmation results until 6 mo after the end of the recruitment phase in each study phase.

We used a pre- and post-intervention study design to evaluate the ability of a simplified HIV test and treat intervention to reduce mortality among newly diagnosed HIV/AIDS cases. The original design was one pre-intervention and one post-intervention phase. The design was modified to have two pre-intervention and two post-intervention phases (Fig 1) based on suggestions from peer review. Data from the “pre-intervention 2010” phase, the period from 1 January 2010 to 31 December 2010, and the “pre-intervention 2011” phase, the period from 1 January 2011 to 31 December 2011, were analyzed as the control arm, in comparison to the “post-intervention 2012” phase, the period from 1 July 2012 to 30 June 2013, and the “post-intervention 2013” phase, the period from 1 July 2013 to 30 June 2014. The period from 1 January 2012 to 30 June 2012 was treated as the “intervention transition period.”

### Study Site

Guangxi Zhuang Autonomous Region is one of the provinces in China most heavily affected by HIV/AIDS. In 2011, the overall mortality of HIV-positive individuals in Guangxi was 6.8% compared to a national average mortality of 5%. The mortality in Guangxi was in the medium-to-high range among the 31 provinces in China. However, Guangxi reported the highest absolute number of HIV-related deaths, accounting for 22% of the deaths in all 31 provinces in China. Late diagnosis (defined as diagnosis at CD4 count ≤ 200 cells/mm^3^ or no CD4 count but clinical AIDS at the time of diagnosis of HIV infection) accounted for approximately one-third of cases in Guangxi from 2007 to 2011. In 2011, nearly 70% of newly diagnosed cases had an initial CD4 count ≤ 350 cells/mm^3^, and among the cases who died in 2011, 48% had been diagnosed in the same calendar year [18]. About 79% of individuals who died of HIV-related causes had never received ART, indicating that linkage to care was suboptimal. Zhongshan County and Pubei County were selected as study sites because they had previously reported high proportions of deaths occurring within the same calendar year of HIV/AIDS diagnosis. In 2011, the respective cumulative numbers of HIV cases in Zhongshan and Pubei were 625 and 986, and the main route of transmission was heterosexual contact. The 2011 mortality was 33.9% and 31.1% of newly diagnosed HIV/AIDS cases in Zhongshan and Pubei, respectively.

### Study Participants

Eligibility criteria were the following: (1) participants were newly diagnosed HIV-positive adults (≥ 18 y), (2) participants received a positive confirmation test at a study site (defined as a WB test that met the national laboratory standards [19]), and (3) participants resided within a study clinic’s catchment area. Participants were followed from the date of HIV confirmation to the end of the study phase, and the follow-up duration for study participants ranged from 6 to 18 mo.

### Standard-of-Care (Pre-Intervention) Procedures

HIV screening in Guangxi is available through health care facilities at the township level and above (i.e., in ascending order, the levels township, county, city, provincial, and national). Screening is available through self-referral for testing and provider-initiated testing (which is routine in surgery departments, sexually transmitted infection clinics, and maternal care clinics). Under China’s national policies, patients receive at least two screening tests in succession (ELISA [enzyme-linked immunosorbent assay] or rapid test) and one WB test to confirm a diagnosis of HIV infection. In the two study counties, patients are screened for HIV using a rapid test at the initiating facility. If the initial screening test is positive, the patient is asked to return to the same facility to give a second blood sample, which is sent to the local county Center for Disease Control and Prevention (CDC) laboratory. This second screening test is analyzed using an ELISA test; if positive, the sample is sent to the city-level CDC laboratory for confirmatory WB testing. About 29%–36% of patients who screen HIV-positive at health care facilities fail to present for the second blood draw, marking the first drop-off in the care cascade. WB results are usually available within 7–18 d, and if the WB test is positive, the patient is asked to provide a third blood sample at the county CDC, which is transferred to the city CDC laboratory for CD4 testing. After the CD4 results are available (typically within 7–18 d), patients eligible for ART (CD4 count ≤ 350 cells/mm^3^) are asked to seek treatment at a separate facility designated to provide ART, which is usually based at the county general hospital [15].

Before treatment initiation, patients are expected to receive education and counseling on ART and adherence, and a physical exam. A fourth blood sample is collected for baseline pre-ART assessment, including kidney function, liver function, and other routine assessments. Patients who are not eligible for ART at diagnosis are advised to undergo CD4 testing every 6 mo for ongoing reassessment of ART eligibility. In many counties, the diagnostic and treatment initiation process requires patients to independently navigate multiple clinic visits, often at different facilities. For patients who are ART-eligible at diagnosis, the usual timeline from the initial screening to ART initiation is 2 to 4 mo. Treatment medication is provided free of charge through the NFATP. Data for the pre-intervention phase were collected through retrospective records review.

### Simplified Test and Treat Intervention Procedures

The simplified test and treat intervention incorporated (1) a streamlined, standardized time frame for diagnosis and (2) expanded access to ART. These changes were enacted under the leadership of the National Center for AIDS/STD Control and Prevention (NCAIDS) at the Chinese Center for Disease Control and Prevention (China CDC), and included reorganization of services [20], with an emphasis on linkage and integration of services. Other aspects of the intervention included provider training and guidelines that accelerated treatment provision [21].

#### Standardized framework for diagnosis: organizational structure and integration of services

The simplified test and treat intervention framework designated activities to take place on a predetermined schedule. Following a positive HIV screening test, the patient received post-screening counseling (e.g., basic education on HIV/AIDS knowledge, the benefits of ART, and the importance of encouraging partners to receive HIV testing) on the same day at the same facility. At this time, the patient was scheduled for a visit at the HIV/AIDS clinic of the county general hospital on the following Wednesday. The HIV/AIDS epidemiologist from the county CDC and the chief physician of the county general hospital’s HIV/AIDS clinic were proactively notified regarding the newly diagnosed patients that were expected to visit the HIV/AIDS clinic the following Wednesday. Every Wednesday, an infectious disease physician and nurses from the HIV/AIDS clinic of the county general hospital carried out medical consultations for ART, blood sample collections for WB confirmation tests and CD4 cell count tests, pretreatment physical examinations, liver and kidney function tests, treatment of opportunistic infections and other co-morbid conditions, and other services as appropriate. The county CDC HIV/AIDS epidemiologist was based at the county general hospital each Wednesday to coordinate the delivery of blood samples from the county general hospital to the city CDC laboratory for same-day WB and CD4 cell count testing and to confirm that patients who screened HIV-positive within the past week presented for their scheduled visit. If a patient failed to arrive at the Wednesday HIV/AIDS clinic visit, the epidemiologist informed the referring clinician to follow-up with the patient and to reschedule the HIV/AIDS clinic visit for the following Wednesday. Every Friday, the city CDC delivered the HIV confirmation and CD4 cell count results from the previous Wednesday’s samples to the county CDC HIV/AIDS epidemiologist, who transferred the results to the county general hospital. The county general hospital was responsible for patient notification and all subsequent follow-up care. The county CDC HIV/AIDS epidemiologist prepared a monthly report on all newly diagnosed HIV cases, testing results, and treatment referral rates.

#### Expanded access to ART: training and guidelines

ART eligibility was expanded to patients with a confirmed HIV diagnosis, irrespective of CD4 cell count, thereby superseding the previous primary criterion for ART initiation of CD4 count ≤ 350 cells/mm^3^. The simplified test and treat intervention explicitly included a minimum of three counseling sessions prior to treatment initiation. The first ART counseling session was provided by the staff of the originating facility immediately after screening. The first counseling session emphasized the importance of presenting to follow-up visits and ensured that the patients had the directions and contact information for the county general hospital designated for providing ART services. The second ART counseling session was provided by the county general hospital physician at the time of collecting blood for HIV confirmatory and CD4 cell count testing. The third ART counseling session was provided when patients received their HIV confirmatory and CD4 cell count results. During each counseling session, all patients were strongly encouraged to initiate ART promptly. Treatment medication was provided free of charge through the NFATP, which is consistent with the standard-of-care procedure.

In order to implement the structural intervention described above, an initial planning meeting was held with key stakeholders, including research staff from NCAIDS/China CDC, officials from the Guangxi Health Department, technical experts from Guangxi CDC, health officials from the Pubei and Zhongshan county health departments, hospital directors and HIV/AIDS clinicians from the two county general hospitals, county CDC directors, and county CDC HIV/AIDS epidemiologists. A consensus was reached on the structural intervention components and the corresponding implementation plan for the two study sites. Policy papers on implementing the simplified test and treat intervention were issued by the two local county health departments and distributed to all health facilities that provide HIV screening in the two counties.

NCAIDS carried out training workshops for providers to review the new HIV care guidelines, practice mock exercises, strengthen communication skills, and reinforce professional expectations. Clinicians who conduct HIV screening tests were also intensively trained on providing post-screening counseling. A handbook was issued to all providers on HIV testing, treatment, and prevention, and educational materials were prepared for distribution to newly diagnosed HIV-positive patients. To monitor the intervention, site supervisors from NCAIDS were stationed in the two study sites for the first 2 mo of the intervention. Afterwards, site visits were conducted monthly to monitor adherence to the intervention protocol. The total cost for the study intervention was approximately US$14,525.75.

### Data Management

The local county CDC HIV/AIDS epidemiologists are legally responsible for following up on all HIV-positive patients to record demographic information, eligibility for ART, and present status (i.e., in regular HIV care, lost to follow-up, migrated out of the county, or deceased) every 6 mo. Physicians providing ART are required to collect ART-related information, including medication side effects and ART status (i.e., engaged on ART, dropped out of ART, lost to follow-up, migrated out of the county, or deceased) every 3 mo and success or failure of ART based on viral load testing once a year. In the pre-intervention phase, per the standard of care, the CDC HIV/AIDS epidemiologists collected data independently of the ART-providing clinicians. In the intervention phase, the HIV/AIDS epidemiologists collaborated with clinicians to record patient data using standardized case report forms. Data were subsequently entered into the national HIV/AIDS case reporting and NFATP databases. These two databases are subsystems of China’s Comprehensive Response Information Management System (CRIMS), a national web-based real-time data system for HIV care and follow-up, which has been previously described [22]. An HIV/AIDS dataset for Pubei and Zhongshan covering 1 January 2010 to 31 December 2014 was downloaded from CRIMS. The study participants in the pre-intervention 2010 phase comprised patients newly diagnosed as HIV-positive between 1 January 2010 and 31 December 2010 and were followed until 30 June 2011. The study participants in the pre-intervention 2011 phase comprised patients newly diagnosed as HIV-positive between 1 January 2011 and 31 December 2011 and were followed until 30 June 2012. The study participants in the post-intervention 2012 phase were patients newly diagnosed as HIV-positive between 1 July 2012 and 30 June 2013 and were followed until 31 December 2013. The study participants in the post-intervention 2013 phase were patients newly diagnosed as HIV-positive between 1 July 2013 and 30 June 2014 and were followed until 31 December 2014. In all four phases, each participant was followed for a duration between 6 and 18 mo. We used data from the 12 mo preceding the pre-intervention 2011 phase and from the 12 mo after the post-intervention 2012 phase to assess potential secular time trends independent of the intervention, based on suggestions from peer review.

### Statistical Analysis

Survival times were calculated as the time from HIV confirmation until death or the last follow-up (at which point survival times were censored). The number of days (median, interquartile range [IQR]) from HIV-positive screening to HIV confirmatory testing, the total number of deaths, and mortality rates were tabulated, stratified by study phase. The overall proportions receiving ART and receiving ART within 30 d of HIV confirmation in each phase were compared by chi-square tests. Kaplan-Meier curves describing survival and ART initiation were compared by log-rank tests. Cox proportional hazards multivariate regressions were used to calculate hazard ratios (HRs) for the effect of the study phase on mortality after adjustment for baseline demographic risk factors. We estimated adjusted hazard ratios (aHRs) and 95% confidence intervals (CIs) from the Cox regressions, using the pre-intervention 2011 phase as a reference group. In addition, we performed an analysis where we further adjusted for variables that could have been modified by the intervention (baseline CD4 or clinical status, and ART initiation). ART initiation was treated as a time-dependent covariate, which took the value zero until the time that a patient first received ART. The proportional hazards assumption for all covariates was assessed by testing the Martingale residuals, and we found no evidence that this assumption was violated. Because the treatment eligibility criteria changed between the pre-intervention and post-intervention study phases, we conducted additional analyses among the subset of individuals with CD4 count ≤ 350 cells/mm^3^ or with clinical AIDS so that the treatment-related outcomes were comparable between the two pre-intervention and two post-intervention phases. Statistical analyses were performed using SAS (version 9.1.3, SAS Institute).

We estimated the actual cost of implementing the intervention, including the cost of the training of local health workers, incentives for health workers for timely referral and initiation of patients on ART, monitoring of the implementation, and educational materials. We excluded any costs that were specific to research. The number of additional patients receiving ART because of the intervention was estimated for the initial year of the intervention (post-intervention 2012 phase) and for the second year of the intervention (post-intervention 2013 phase), based on differences in the ART coverage rate compared to that in the pre-intervention 2011 phase. The number of deaths prevented because of the intervention was estimated for the post-intervention 2012 phase and for the post-intervention 2013 phase, based on the differences in overall mortality compared to that in the pre-intervention 2011 phase. We estimated the cost both as a total for the program and as an incremental cost per incremental patient achieving the primary outcome.

Among the above, the approach to stratifying the proportional hazard assumptions, assessing potential secular trends, and conducting the cost analysis emerged from the review process.

### Ethics

The study protocol was reviewed and approved by the Institutional Review Board of NCAIDS, China CDC (#X120717220). All patients who screened HIV-positive were requested to give their written informed consent for the use of their de-personalized data in future epidemiological analysis. No additional study-specific written informed consent was obtained. None of the participants opted out. There was no incentive for study participants.

## Results

**Fig 2:**
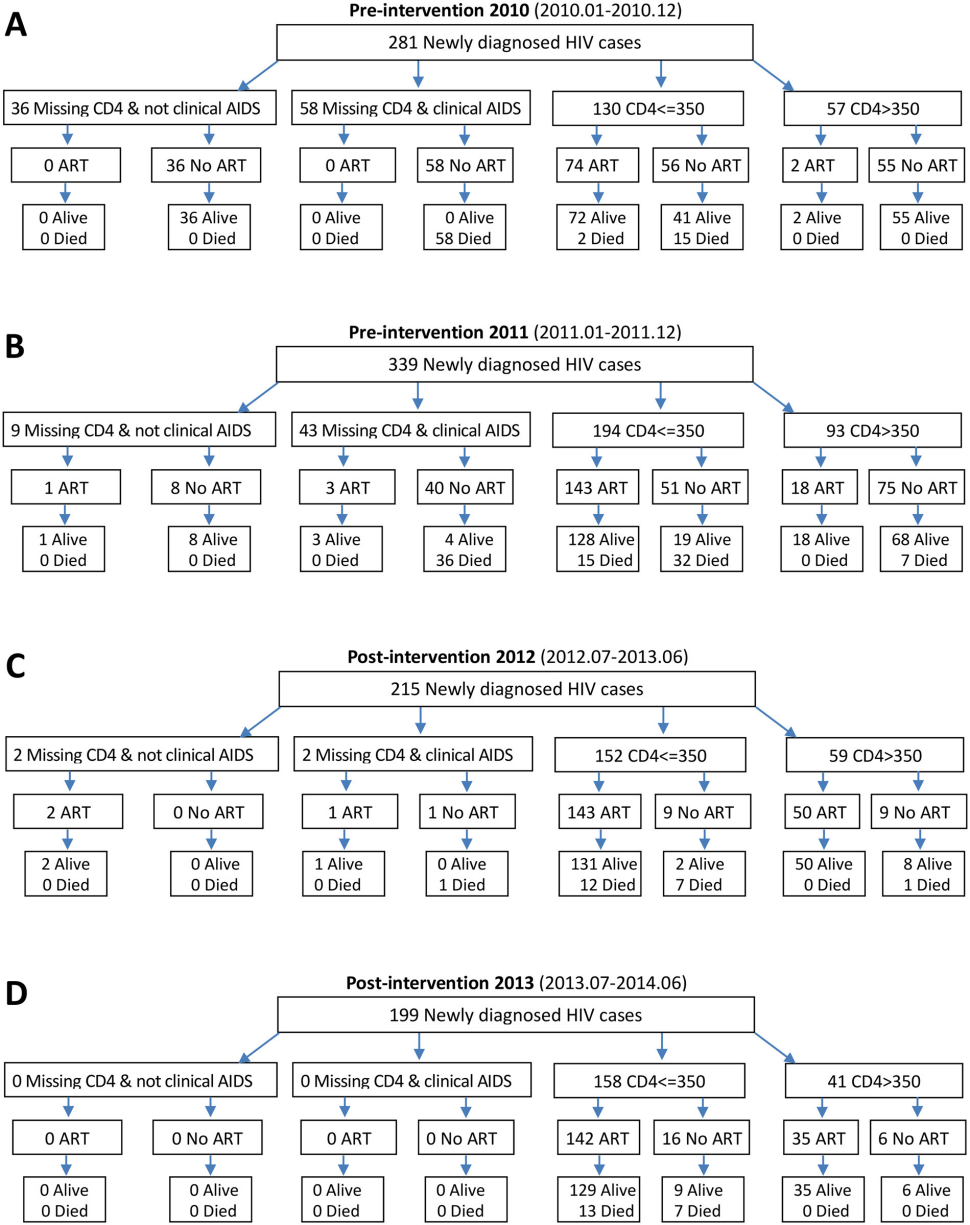
Number of HIV diagnoses, ART initiations, and deaths in the pre-intervention 2010, pre-intervention 2011, post-intervention 2012, and post-intervention 2013 phases in Guangxi, China.

As shown in Fig 2, a total of 281 and 339 newly diagnosed HIV-positive patients were included in the pre-intervention 2010 and pre-intervention 2011 phases, respectively, and 215 and 199 patients in the post-intervention 2012 and post-intervention 2013 phases, respectively. Among the 281 patients in the pre-intervention 2010 phase, 76 (27.0%) enrolled in ART, and 75 (26.7%) died, including two deaths following ART initiation. Among the 339 participants in the pre-intervention 2011 phase, 165 (48.7%) enrolled in ART, and 90 (26.5%) died, including 15 deaths following ART initiation. During the post-intervention 2012 phase, 196 out of 215 (91.2%) individuals enrolled in ART, and 21 (9.8%) individuals died, including 12 deaths following ART initiation. During the post-intervention 2013 phase, 177 out of 199 (89%) patients enrolled in ART, and 20 (10.0%) patients died, including 13 deaths following ART initiation. Median participant follow-up was 8.77 mo (IQR 6.0–12.2) for the post-intervention 2013 phase and 9.2 mo (IQR 5.9–12.2) for the post-intervention 2012 phase, compared to 7.9 mo (IQR 5.6–11.2) for the pre-intervention 2011 phase and 7.33 mo (IQR 4.1–11.1) for the pre-intervention 2010 phase. In the intervention transition period (not included in Fig 2), 111 patients were diagnosed with HIV infection, 77 (69.4%) enrolled in ART, and 22 died (19.8%), including seven deaths following ART initiation.

Of the total 1,034 participants (Table 1), 746 (72%) were male, and the median age was 48 y (IQR 36–60). Nearly all participants had a middle school education or below (95.6%) and were employed as farmers (90.6%). Over half of the individuals were married or partnered (56.5%). The participants who were enrolled during the four phases were not statistically significantly different on any baseline demographic characteristics. The predominant mode of HIV transmission was heterosexual contact, and this remained consistent throughout the study. Among 111 patients diagnosed in the intervention transition period (not included in Table 1), 87 (78.4%) were male, median age was 51 y (IQR 38–60), and 109 cases were infected by heterosexual transmission, and two cases via injecting drug use.

**Table 1:** Characteristics of newly diagnosed HIV cases in the pre-intervention 2010, pre-intervention 2011, post-intervention 2012, and post-intervention 2013 phases in Guangxi, China.

Characteristic	Pre-Intervention 2010 (Jan 2010–Dec 2010), *n* = 281 (1)		Pre-Intervention 2011 (Jan 2011–Dec 2011), *n* = 339 (2)		Post-Intervention 2012 (Jul 2012–Jun 2013), *n* = 215 (3)		Post-Intervention 2013 (Jul 2013–Jun 2014), *n* = 199 (4)		*p*-Value (1) versus (2)	*p*-Value (2) versus (3)	*p*-Value (3) versus (4)
	*n* or Median	Percent or IQR	*n* or Median	Percent or IQR	*n* or Median	Percent or IQR	*n* or Median	Percent or IQR			
***Demographic variables***											
**Gender**									0.210	0.532	0.742
Male	195	69%	251	74%	154	71%	146	73%			
Female	86	31%	88	26%	61	29%	53	27%			
**Age (years)**	46	31–62	52	33–63	46	37–58	49	37–59	0.131	0.281	0.446
**Mode of HIV infection**									0.708	0.656	0.342
Heterosexual contact	266	95%	324	96%	208	97%	196	98%			
Other	15	5%	15	4%	7	3%	3	2%			
**Educational attainment**									0.120	0.813	0.632
Middle school or below	264	94%	328	97%	207	96%	189	95%			
High school or above	17	6%	11	3%	8	4%	10	5%			
**Occupation**									0.445	0.373	0.319
Farmer	246	88%	304	90%	198	92%	189	95%			
Other	35	12%	35	10%	17	8%	10	5%			
**Marital status**									0.255	0.136	0.164
Single, divorced, or widowed	116	41%	156	46%	85	40%	93	47%			
Married or partnered	165	59%	183	54%	130	60%	106	53%			
***Outcome variables***											
**Median baseline CD4 count (cells/mm** ^**3**^ **)**	243	75–384	219	77–403	220	69–379	178	53–330	0.672	0.462	0.078
**CD4 subgroups**									<0.001	<0.001	—
≤200 cells/mm^3^	80	28%	135	40%	100	47%	111	56%	<0.001	0.008	0.005
201–350 cells/mm^3^	50	18%	59	17%	52	24%	47	24%	0.723	0.005	0.949
>350 cells/mm^3^	57	20%	93	27%	59	27%	41	21%	0.011	0.857	0.020
Missing CD4 and reported as AIDS case	36	13%	9	3%	2	1%	0	0%	0.009	<0.001	
Missing CD4 and reported as non-AIDS case	58	21%	43	13%	2	1%	0	0%			
**Time from HIV confirmation to CD4 testing (days)**	28	11–145	14	6–42	1	0–4	0	0–-1	<0.001	<0.001	<0.001
**Number of patients with CD4 testing within 30 d of HIV confirmation**	189	67%	205	60.5%	210	97.7%	193	97%	0.080	<0.001	0.663
**Number of ART initiations (regardless of CD4 cell count)**	76	27%	165	48.7%	196	91.2%	177	89%	<0.001	<0.001	0.511
**Number of ART initiations within 30 d of HIV confirmation**	24	9%	68	20.1%	175	81.4%	160	80%	<0.001	<0.001	0.707
**Time from HIV screening to HIV confirmation (days) for ART-initiated patients**	11	6–22	11	7–18	7	3–10	6	3–9	0.896	<0.001	0.923
**Time from HIV confirmation to ART initiation (days) for ART-initiated patients**	53	27–141	43	15–113	5	2–12	5	2–13	0.036	<0.001	0.823
**Overall mortality**	75	26.7% (75/281)	90	26.5% (90/339)	21	9.8% (21/215)	20	10.1% (20/199)	0.968	<0.001	0.923
Mortality of ART-initiated patients	2	2.6% (2/76)	15	9.1% (15/165)	12	6.1% (12/196)	13	7.3% (13/177)	0.050	0.286	0.526
Mortality of untreated patients	73	35.6% (73/205)	75	43.1% (75/174)	9	47.4% (9/19)	7	31.8% (7/22)	0.030	0.722	0.145
**Number of ART initiations (CD4 ≤ 350 cells/mm** ^**3**^ **or missing CD4 but reported as AIDS case)**	74	39.4% (74/188)	146	61.6% (146/237)	144	93.5% (144/154)	142	89.9% (142/158)	<0.001	<0.001	0.246
**Mortality of patients with CD4 ≤ 350 cells/mm** ^**3**^ **or missing CD4 but reported as AIDS case**	75	39.9% (75/188)	83	35.0% (83/237)	20	13.0% (20/154)	20	12.7% (20/158)	0.160	<0.001	0.915
Mortality of ART-initiated patients	2	2.7% (2/74)	15	10.3% (15/146)	12	8.3% (12/144)	13	9.2% (13/142)	0.032	0.108	0.719
Mortality of untreated patients	73	64.0% (73/114)	68	74.7% (68/91)	8	80.0% (8/10)	7	43.8% (7/16)	0.009	<0.001	0.021

In the pre-intervention 2010 and pre-intervention 2011 phases, 67% (median CD4 count = 243 cells/mm^3^, IQR 75–384) and 60.5% (median CD4 count = 219 cells/mm^3^, IQR 77–403) of participants had a baseline CD4 cell count within 30 d of HIV confirmation, respectively. This value was statistically significantly higher in the post-intervention 2012 phase (97.7%, median CD4 count = 220 cells/mm^3^, IQR 69–379, all *p* < 0.001[i.e., for all comparisons between a pre- and post-intervention phase]) and in the post-intervention 2013 phase (97%, median CD4 count = 178 cells/mm^3^, IQR 53–330, all *p* < 0.001). In the pre-intervention 2010 and pre-intervention 2011 phases, 34% and 16% of patients failed to obtain CD4 testing at any time point, compared to 2% and 0% in the post-intervention 2012 and post-intervention 2013 phases (all *p* < 0.001), respectively. The median time from HIV confirmatory testing to CD4 testing was 28 d (IQR 11–145) in the pre-intervention 2010 phase and 14 d (IQR 6–42) in the pre-intervention 2011 phase, compared to 1 d (IQR 0–4) in the post-intervention 2012 phase (all *p* < 0.001) and 0 d (IQR 0–1) in the post-intervention 2013 phase (all *p* < 0.001). In the intervention transition period (not included in Table 1), 93 (83.8%, median CD4 count = 264 cells/mm^3^, IQR 75–433) had a baseline CD4 cell count within 30 d of HIV confirmation, and 11 (10%) of patients failed to obtain CD4 testing at any time point. The median time from HIV confirmatory testing to CD4 testing was 6 d (IQR 4–13).

In the pre-intervention 2010 and pre-intervention 2011 phases, 27% and 48.7% of the total participants initiated ART, respectively, compared to 91.2% and 89% in the post-intervention 2012 and post-intervention 2013 phases, respectively (all *p* < 0.001). Among individuals with CD4 count ≤ 350 cells/mm^3^ or missing their CD4 count but reported as diagnosed with AIDS, 39.4% in the pre-intervention 2010 phase and 61.6% in the pre-intervention 2011 phase initiated ART, compared to 93.5% in the post-intervention 2012 phase and 89.9% in post-intervention 2013 phase (all *p* < 0.001). For patients who initiated ART, the median time from HIV screening to confirmatory testing was 11 d (IQR 6–22) in the pre-intervention 2010 phase and 11 d (IQR 7–18) in the pre-intervention 2011 phase, compared to 7 d (IQR 3–10) in the post-intervention 2012 phase and 6 d (IQR 3–9) in the post-intervention 2013 phase (all *p* < 0.001). The median time from HIV confirmatory testing to treatment initiation was 53 d (IQR 27–141) in the pre-intervention 2010 phase and 43 d (IQR 15–113) in the pre-intervention 2011 phase, compared to 5 d (IQR 2–12) in the post-intervention 2012 phase and 5 d (IQR 2–13) in the post-intervention 2013 phase (all *p* < 0.001). In the intervention transition period (not included in Table 1), 77 cases (69.4%) initiated ART, and the median time from HIV confirmatory testing to treatment initiation was 25 d (IQR 7–97).

A total of 228 deaths occurred over the entire study observation period, with a crude mortality of 26.7% in the pre-intervention 2010 phase and 26.5% in the pre-intervention 2011 phase, compared to 9.8% in the post-intervention 2012 phase and 10.1% in the post-intervention 2013 phase (all *p* < 0.001) and 19.8% in the intervention transition period (not included in Table 1). Among individuals with CD4 count ≤ 350 cells/mm^3^ or missing CD4 count but diagnosed with AIDS, 198 deaths occurred, with mortality of 39.9% in the pre-intervention 2010 phase and 35.0% in the pre-intervention 2011 phase, compared to 13.0% in the post-intervention 2012 phase and 12.7% in the post-intervention 2013 phase (all *p* < 0.001). Reassuringly, the primary outcome variable of overall mortality was quite consistent within the two pre-intervention phases, at 26.7% and 26.5%, respectively. Mortality was 9.8% and 10.1% in the two post-intervention phases, respectively. We observed 22 deaths among 113 HIV cases newly diagnosed during the 6 mo of the intervention transition period, with a crude mortality of 19.5% (22/113), which falls in between the values of the two pre-intervention phases and the two post-intervention phases. We also calculated the mortality rates for the two pre-intervention phases, the intervention transition period, and the two post-intervention phases, and there were 3.5, 3.4, 2.5, 1.2, and 1.2 deaths/100 person-years, respectively.

**Fig 3:**
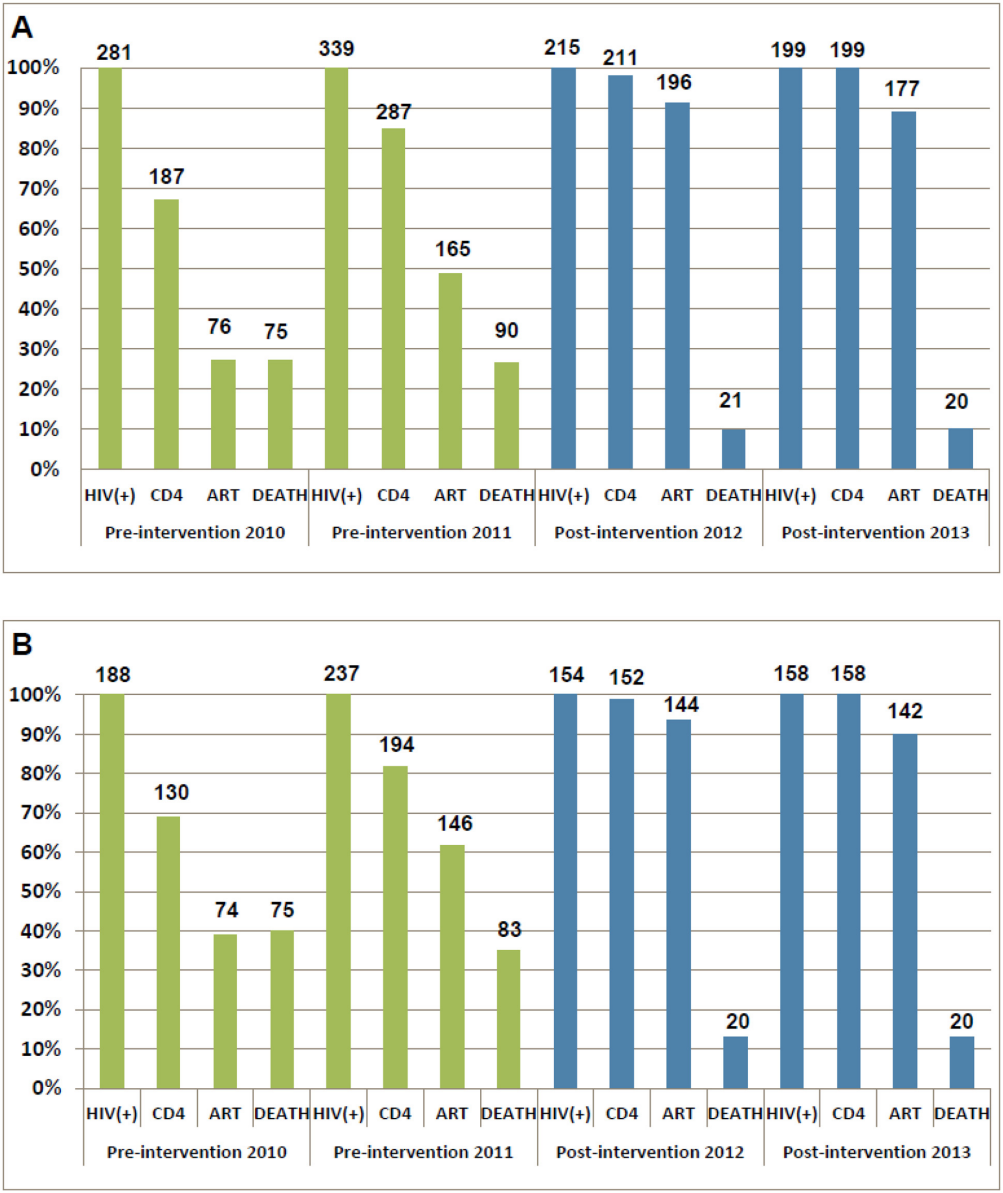
Cascade of confirmed HIV diagnosis, CD4 testing, ART initiation, and mortality during the pre-intervention 2010, pre-intervention 2011, post-intervention 2012, and post-intervention 2013 phases in Guangxi, China. (A) For all HIV cases. (B) For individuals with CD4 count ≤ 350 cells/mm^3^ or missing CD4 but reported as AIDS cases.

The HIV care cascades by study phase are shown in Fig 3. Our study focused on the period between HIV-positive confirmation and death. Among all patients in the pre-intervention 2010 and pre-intervention 2011 phases, 33.5% and 15.3% of patients were lost to follow-up between HIV diagnosis and CD4 testing, respectively. A further 39.5% and 36.0% of patients were lost before ART initiation, respectively, and 26.7% and 26.5% of patients died, respectively. In contrast, in the post-intervention 2012 and post-intervention 2013 phases, only 1.9% and 0.0% of patients were lost to follow-up between HIV diagnosis and CD4 testing, respectively; an additional 7.0% and 11.1% of patients were lost before ART initiation, respectively, and 9.8% and 10.1% of patients died, respectively. Among individuals with CD4 count ≤ 350 cells/mm^3^ or missing CD4 but diagnosed with AIDS, 30.9% of patients in the pre-intervention 2010 phase and 18.1% of patients in the pre-intervention 2011 phase were lost to follow-up between HIV diagnosis and CD4 testing, a further 29.8% and 20.3% were lost before ART initiation, respectively, and 39.9% and 35.0% died, respectively. Among comparable patients in the post-intervention 2012 and post-intervention 2013 phases, 1.3% and 0.0% were lost to follow-up between HIV diagnosis and CD4 testing, respectively, a further 5.2% and 10.1% were lost before ART initiation, respectively, and 13.0% and 12.7% died, respectively. In the intervention transition period (not included in Fig 3), among all newly diagnosed cases, 9.9% of patients were lost to follow-up between HIV diagnosis and CD4 testing, a further 20.7% were lost before ART initiation, and 19.8% died. Among individuals with CD4 count ≤ 350 cells/mm^3^ or missing CD4 but diagnosed with AIDS, 12.9% of patients were lost to follow-up between HIV diagnosis and CD4 testing, a further 11.4% were lost before ART initiation, and 31.4% died.

**Fig 4:**
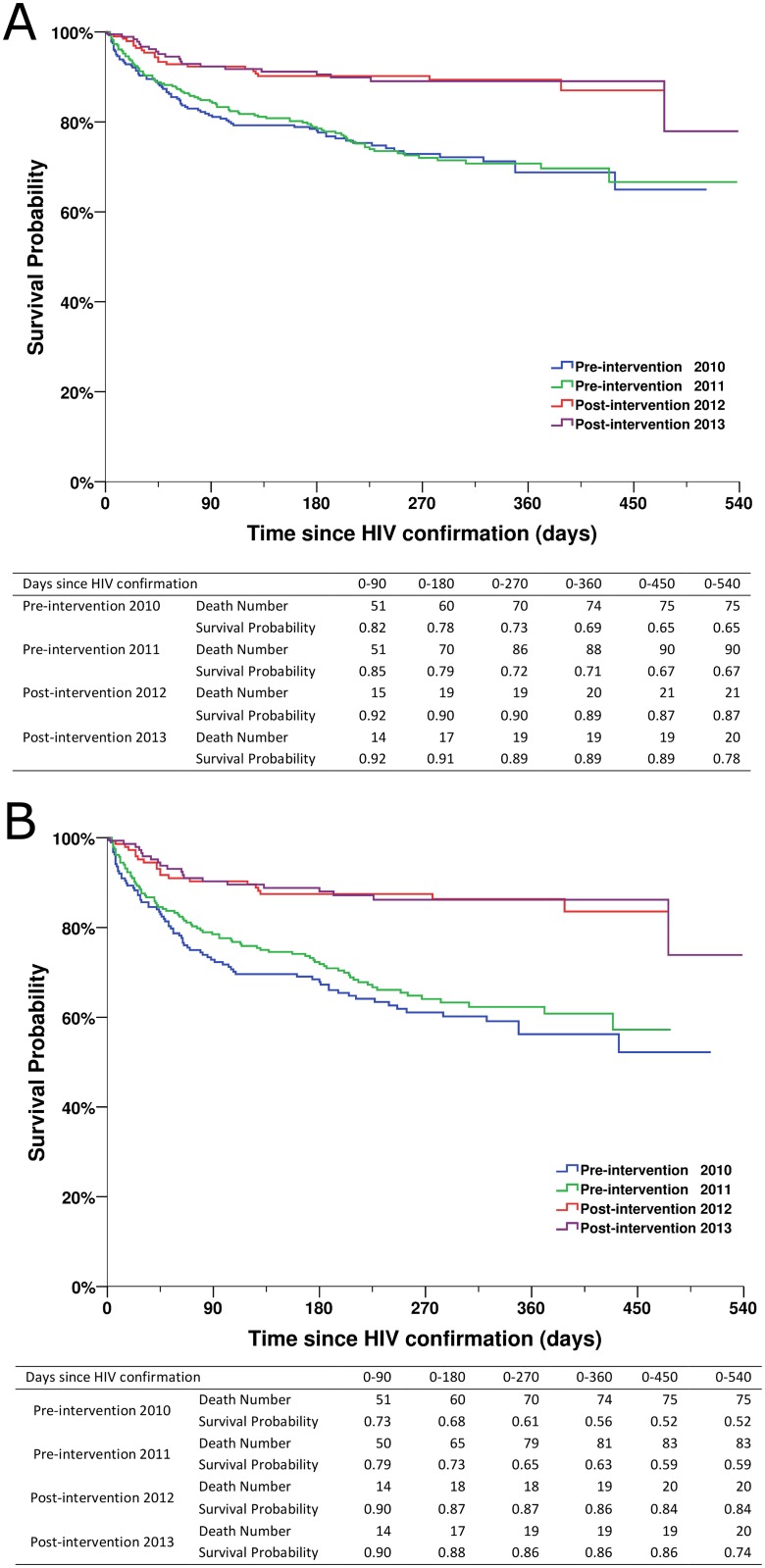
Kaplan-Meier survival curves for newly diagnosed HIV cases in the pre-intervention 2010, pre-intervention 2011, post-intervention 2012, and post-intervention 2013 phases in Guangxi, China. (A) For all HIV cases. (B) For individuals with CD4 count ≤ 350 cells/mm^3^ or missing CD4 but reported as AIDS cases.

**Fig 5:**
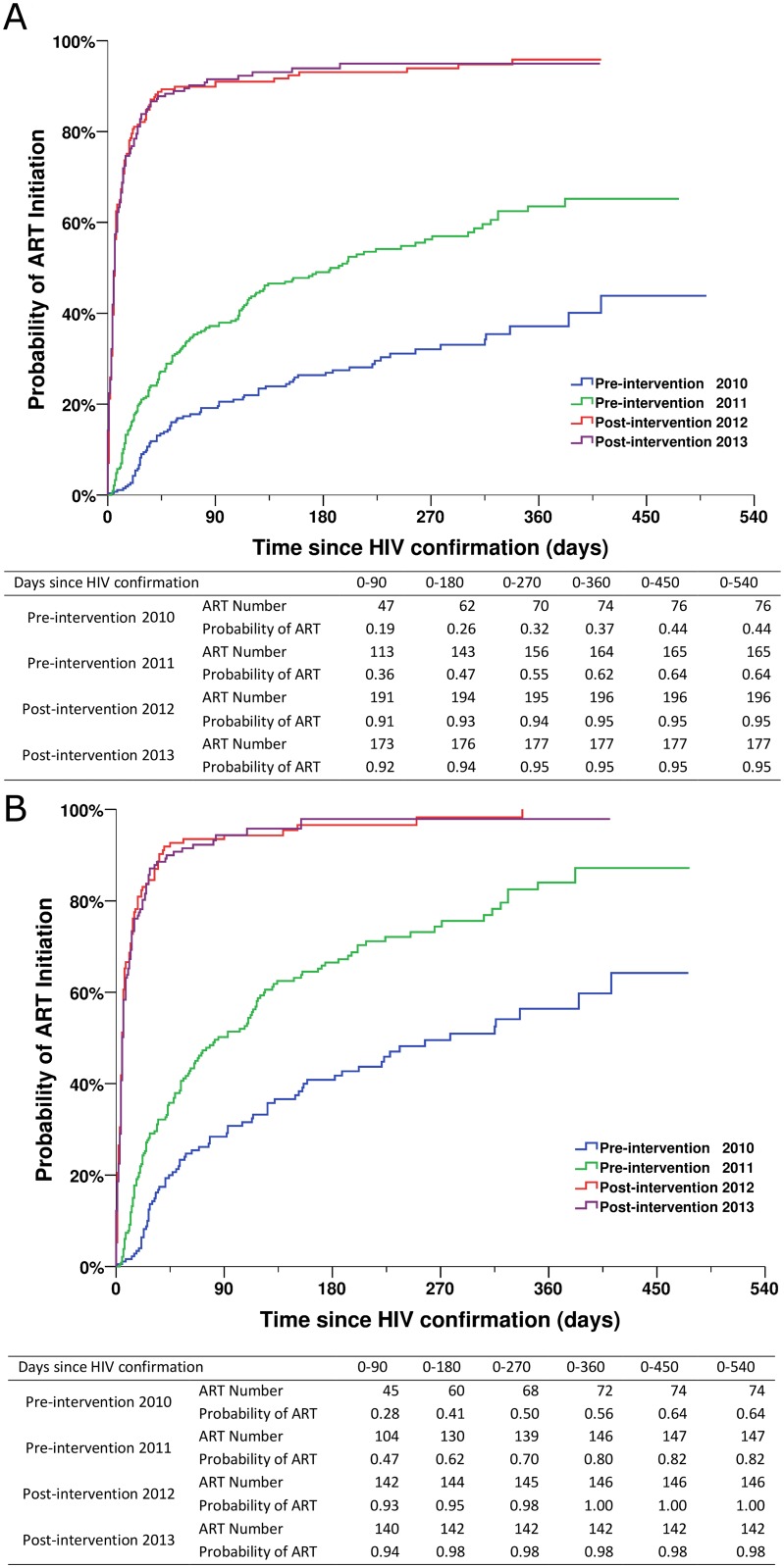
Kaplan-Meier curves for ART initiation for newly diagnosed HIV cases in the pre-intervention 2010, pre-intervention 2011, post-intervention 2012, and post-intervention 2013 phases in Guangxi, China. (A) For all HIV cases. (B) For individuals with CD4 count ≤ 350 cells/mm^3^ or missing CD4 but reported as AIDS cases.

Patients in the two post-intervention phases had significantly higher survival rates over the follow-up period than those in the two pre-intervention phases (all *p* < 0.001; Fig 4). As shown in Fig 5, within 90 d of HIV confirmation, ART coverage in the post-intervention 2012 and post-intervention 2013 phases was similar and was 2.5 times the coverage in the pre-intervention 2011 phase and 4.8 times the coverage in the pre-intervention 2010 phase for all newly diagnosed patients, and 2.0 times and 3.3 times, respectively, for patients with CD4 count ≤ 350 cells/mm^3^ or missing CD4 but reported as AIDS cases.

Table 2 describes factors associated with mortality for study participants pooled from the two pre-intervention and the two post-intervention phases. In univariate analysis, factors associated with death were being male (HR 2.604, 95% CI 1.758–3.856, *p* < 0.001), every 10-y increase in age (HR 1.258, 95% CI 1.155–1.371), CD4 count ≤ 200 cells/mm^3^ or AIDS (HR 15.344, 95% CI 8.918–26.402, *p* < 0.001), and receiving ART (HR 0.231, 95% CI 0.162–0.328, *p* < 0.001). The simplified test and treat intervention was statistically strongly associated with a decreased risk for mortality (HR 0.361, 95% CI 0.224–0.580, *p* < 0.001, for post-intervention 2012; HR 0.367, 95% CI 0.226–0.603, *p* < 0.001, for post-intervention 2013).

**Table 2:** Mortality among all newly diagnosed HIV cases during the pre-intervention 2010, pre-intervention 2011, post-intervention 2012, and post-intervention 2013 phases, based on Cox model analysis.

Characteristic	Number of Deaths	Observed Person- Months	Mortality Rate (Events/100 Person-Months)	HR (95% CI)	*p*-Value	aHR-1 (95% CI)	*p*-Value	aHR-2 (95% CI)	*p*-Value
**Study phase**									
Pre-intervention 2010 (*n* = 281)	75	2,113	3.5	1.025 (0.754–1.392)	0.877	1.137 (0.835–1.547)	0.415	0.952 (0.697–1.301)	0.758
Pre-intervention 2011 (*n* = 339)	90	2,629	3.4	1		1		1	
Post-intervention 2012 (*n* = 215)	21	1,816	1.2	0.361 (0.224–0.580)	<0.001	0.385 (0.239–0.620)	<0.001	1.034 (0.611–1.752)	0.900
Post-intervention 2013 (*n* = 199)	20	1,650	1.2	0.367 (0.226–0.603)	<0.001	0.380 (0.233–0.618)	<0.001	0.882 (0.517–1.504)	0.644
**Gender**									
Female	29	2,540	1.1	1		1		1	
Male	177	5,668	3.1	2.604 (1.758–3.856)	<0.001	2.552 (1.720–3.786)	<0.001	1.952 (1.314–2.898)	0.001
**Every 10-y increase in age**	206	8,209	2.5	1.258 (1.155–1.371)	<0.001	1.211 (1.115–1.315)	<0.001	1.132 (1.035–1.238)	0.007
**Baseline CD4 count or clinical status**									
CD4 ≤ 200 cells/mm^3^ or AIDS	192	3,754	5.1	15.344 (8.918–26.402)	<0.001			19.286 (11.132–33.414)	<0.001
CD4 > 200 cells/mm^3^ or non-AIDS	14	4,455	0.3	1				1	
**ART initiation status**									
Yes	42	5,590	0.8	0.231 (0.162–0.328)	<0.001			0.139 (0.089–0.217)	<0.001
No	164	2,618	6.3	1				1	

HR: HR based on univariate Cox model; aHR-1: aHR based on multivariate Cox model adjusted for demographic variables (age and gender), but not adjusted for intervention-changeable variables (baseline CD4 count or clinical status, ART initiation); aHR-2: aHR based on multivariate Cox model adjusted for both demographic variables (age and gender) and intervention-changeable variables (baseline CD4 count or clinical status, ART initiation).

In the multivariate proportional hazards model, after adjusting for age and gender, the simplified test and treat intervention was significantly associated with decreased mortality compared to pre-intervention 2011 (aHR 0.385, 95% CI 0.239–0.620, *p* < 0.001, for post-intervention 2012; aHR 0.380, 95% CI 0.233–0.618, *p* < 0.001, for post-intervention 2013). When the variables that were potentially modified by the intervention were also included in the regression model (i.e., CD4 testing, CD4 cell counts, and ART initiation), we found that the intervention was no longer significantly associated with mortality (aHR 1.034, 95% CI 0.611–1.752, *p* = 0.900, for post-intervention 2012; aHR 0.882, 95% CI 0.517–1.504, *p* = 0.644, for post-intervention 2013). This result suggests that the impact of the intervention on mortality was meditated through changes in these other variables.

We repeated the analyses for the subset of individuals with CD4 count ≤ 350 cells/mm^3^ or clinical AIDS (Table 3). Under the multivariable model that controlled for gender and age, the simplified test and treat intervention was significantly associated with reduced mortality compared to pre-intervention 2011 (aHR 0.369, 95% CI 0.226–0.603, *p* < 0.001, for post-intervention 2012; aHR = 0.361, 95% CI 0.221–0.590, *p* < 0.001, for post-intervention 2013). As was the case for all HIV cases, after adjustment for variables that were specifically changed by the intervention, the intervention’s effect on mortality was no longer statistically significant (aHR 1.033, 95% CI 0.601–1.774, *p* = 0.907, for post-intervention 2012; aHR 0.915, 95% CI 0.534–1.567, *p* = 0.746, for post-intervention 2013).

**Table 3:** Mortality among newly diagnosed treatment-eligible HIV cases (with CD4 count ≤ 350 cells/mm^3^ or missing CD4 but reported as AIDS cases) during the pre-intervention 2010, pre-intervention 2011, post-intervention 2012, and post-intervention 2013 phases, based on Cox model analysis.

Characteristic	Number of Deaths	Observed Person- Months	Mortality Rate (Events/100 Person-Months)	HR (95% CI)	*p*-Value	aHR-1 (95% CI)	*p*-Value	aHR-2 (95% CI)	*p*-Value
**Study phase**									
Pre-intervention 2010 (*n* = 188)	75	1,363	5.5	1.160 (0.849–1.585)	0.353	1.293 (0.944–1.772)	0.110	1.036 (0.753–1.424)	0.829
Pre-intervention 2011 (*n* = 237)	83	1,746	4.8	1		1		1	
Post-intervention 2012 (*n* = 154)	20	1,332	1.5	0.353 (0.211–0.560)	<0.001	0.369 (0.226–0.603)	<0.001	1.033 (0.601–1.774)	0.907
Post-intervention 2013 (*n* = 158)	20	1,282	1.6	0.344 (0.211–0.561)	<0.001	0.361 (0.221–0.590)	<0.001	0.915 (0.534–1.567)	0.746
**Gender**									
Female	28	1,646	1.7	1		1		1	
Male	170	4,077	4.2	2.312 (1.550–3.449)	<0.001	2.384 (1.596–3.562)	<0.001	1.915 (1.280–2.866)	0.002
**Every 10-y increase in age**	198	5,723	3.5	1.194 (1.047–1.361)	<0.001	1.166 (1.066–1.275)	0.001	1.128 (1.028–1.237)	0.011
**Baseline CD4 count or clinical status**									
CD4 ≤ 200 cells/mm^3^ or AIDS	192	3,754	5.1	15.405 (6.834–34.725)	<0.001			15.116 (6.696–34.176)	<0.001
CD4 > 200 cells/mm^3^ or non-AIDS	6	1,970	0.3	1				1	
**ART initiation**									
Yes	42	4,658	0.9	0.154 (0.108–0.221)				0.144 (0.092–0.225)	
No	156	1,065	14.6	1	<0.001			1	<0.001

HR: HR based on univariate Cox model; aHR-1: aHR based on multivariate Cox model adjusted for demographic variables (age and gender), but not adjusted for intervention-changeable variables (baseline CD4 or clinical status, ART initiation); aHR-2: aHR based on multivariate Cox model adjusted for both demographic variables (age and gender) and intervention-changeable variables (baseline CD4 or clinical status, ART initiation).

The cost analysis results are shown in Table 4. The total cost of the simplified test and treat intervention implementation was US$14,525.75, with a cost of US$13,750.00 and US$775.75 for the first and second year, respectively. The unit cost for an additional patient receiving ART attributable to the intervention was US$83.80, with a cost of US$147.46 and US$9.69 for the first and second year, respectively (Table 5). The unit cost of a death prevented because of the intervention was US$234.52, with US$420.08 in the initial year and US$26.56 in the second year.

**Table 4:** Cost analysis of implementation of the intervention in Guangxi, China, 2012–2014.

Variable	Unit Cost	Post-Intervention 2012		Post-Intervention 2013		Total Cost
		Number of Units or Individuals	Cost	Number of Units or Individuals	Cost	
**Training workshop for local health workers (1 d/county)**						
Three trainers: ground transportation	16.13	3 × 2 person-trips	96.77			
Three trainers: per diem/hotel	69.35	3 × 2 person-days	416.13			
80 health workers in Pubei	29.03	80	2,322.58			
92 health workers in Zhongshan	29.03	92	2,670.97			
Subtotal			5,506.45			5,506.45
**Monthly monitoring by provincial staff (two staff/visit, 4 d/visit)**						
Ground transportation	16.13	2 × 7 person-trips	225.81	2 × 4 person-trips	20.81	
Per diem/hotel	69.35	2 × 28 person-days	3,883.87	2 × 16 person-days	357.96	
Subtotal			4,109.68		378.77	4,488.45
**Training/educational materials**						
Handbook for health workers	1.61	300	483.87			
Educational materials for patients	0.50	2,000	1,000.00			
Subtotal			1,483.87			
**Health worker incentives**						
Referring a patient to ART	8.06	211	1,701.61	199	258.84	
Initiating a patient on ART	4.84	196	948.39	177	138.14	
Subtotal			2,650.00		396.98	3,046.98
**Total**			13,750.00		775.75	14,525.75

All costs are in US dollars.

**Table 5:** Unit cost per patient receiving ART, unit cost per additional patient receiving ART because of the intervention, and unit cost per death prevented because of intervention in Guangxi, China, 2012–2014.

Variable	Post-Intervention 2012 (Total Cost = 13,750.00)		Post-Intervention 2013 (Total Cost = 775.75)		Average Unit Cost
	Number of Patients or Deaths	Unit Cost	Number of Patients or Deaths	Unit Cost	
Patients receiving ART	196	70.15	177	4.38	38.94
Additional patients receiving ART because of intervention	93	147.46	80	9.69	83.80
Deaths prevented because of intervention	33	420.08	29	26.56	234.52

All costs are in US dollars.

## Discussion

Our results show that a simplified HIV test and treat intervention incorporating a streamlined, standardized time frame for diagnosis and expanded access to ART, irrespective of CD4 cell count, resulted in a significant increase in ART coverage within 90 d of diagnosis of HIV infection, from below 36% to over 90% among all newly diagnosed HIV/AIDS cases, and from under 47% to over 93% among newly diagnosed cases with CD4 count ≤ 350 cells/mm^3^ or missing CD4 but reported as AIDS cases. The simplified HIV test and treat intervention was also associated with significantly reduced overall mortality, from about 26% to fewer than 10%.

Our results also show that the cost of the intervention was quite low, and mostly accrued in the initial year, as we set up the intervention. The unit cost per additional patient receiving ART declined to US$9.69 in the second year. Similarly, the unit cost per death prevented attributable to the intervention was US$234.52 over the study period, and it declined to US$26.56 in the second year of the study. Based on these results, we feel that the simplified test and treat intervention represents an effective and sustainable structural intervention that requires very little further investment once it is set up.

China is committed to providing universal access to HIV testing and treatment in concordance with the WHO Treatment 2.0 strategy [1]. However, optimizing the cascade of HIV care, particularly during pre-treatment follow-up and monitoring, remains a key challenge. To address this issue, NCAIDS, China CDC, prioritized the identification, implementation, and assessment of new strategies for improved engagement in HIV testing and treatment.

Few other studies have addressed the test and treat strategy in Asia. Our findings show that the implementation of a simplified test and treat intervention was associated with a significant decrease in mortality rate. Furthermore, this is the first study to our knowledge to show that a structural intervention to streamline HIV testing procedures and to expand treatment eligibility can have a substantial impact on the full HIV care cascade and patient mortality. We present evidence that a reconceptualization of HIV testing and treatment policies can have a dramatic effect on patient outcomes, even in the absence of new diagnostic technologies. Our findings provide program-based evidence to support widespread implementation of this intervention in China and to support similar test and treat strategies elsewhere. Given the size of China’s HIV-positive population, the simplified test and treat intervention at scale could streamline the diagnostic process for hundreds of thousands of patients.

Over the pre-intervention phases, 33%–40% of patients failed to complete CD4 testing within 30 d of HIV confirmation. This is concerning because the CD4 count is the primary determinant of ART eligibility under the standard of care. While the proportion of patients who had CD4 count > 350 cells/mm^3^ remained consistent throughout the study (27%), we noted a higher proportion of patients who had CD4 count ≤ 200 cells/mm^3^ or who were reported as AIDS cases during the pre-intervention phases. This suggests that under the standard-of-care practice (i.e., the pre-intervention study phases), patients who failed to complete CD4 testing were more likely to have lower CD4 cell counts. These results reinforce previous findings that late HIV diagnosis remains a crucial challenge for HIV care providers in China and globally [14,23–25].

Complex testing procedures increase the risk for LTFU. Under the standard of care, the steps of HIV testing and treatment initiation were not co-located. Rather, patients often had to attend several medical facilities, such as the original facility that provided screening (e.g., township clinic), the local CDC, and the county hospital, in order to confirm the diagnosis and to begin treatment. This promotes structural delays in the initiation of treatment and increases the likelihood of attrition. The simplified test and treat intervention provided an opportunity to streamline the procedures from a patient-centered perspective. After the initial screening, all care visits occurred at a single location, the county general hospital. Furthermore, providing concurrent CD4 and WB confirmatory testing reduced the total number of visits, and thus delays, before beginning ART. Our data suggest that the simplified test and treat intervention had significant overall success in expanding access to CD4 testing and promoting initiation of ART, with the consequent favorable impact of decreased mortality.

A small proportion of patients (8.8%–11%) in the two post-intervention phases still failed to initiate treatment, which may be due to stigma, competing needs, insufficient understanding of ART’s benefits, or voluntary refusal of treatment [6,26–29]. To minimize this proportion, the simplified test and treat intervention has multiple built-in opportunities during the pre-treatment process to deliver pre-ART counseling and to encourage ART engagement. In our study, the overwhelming majority of patients were willing to initiate ART, irrespective of CD4 cell count. The initiation of ART for all HIV/AIDS cases is likely to be facilitated in the future as emerging global guidelines evolve to embrace the recently released results of the TEMPRANO [30] and START [31] studies, which definitively confirmed that immediate initiation of ART, irrespective of CD4 cell count, is associated with significant reductions in clinical disease progression [32].

We noted that the number of newly diagnosed patients in the two pre-intervention phases (281 and 339) was higher than the number in the two post-intervention phases (215 and 199) and that the mortality in the two pre-intervention phases (26.7% and 26.5%) was also higher than in the two post-intervention phases (9.8% and 10.1%). The number of people who are diagnosed per year depends on the regional HIV incidence, the performance of HIV testing programs, and individual willingness to be tested. In 2010 (immediately prior to the pre-intervention 2010 phase), Guangxi launched a province-wide 5-y public campaign to promote HIV testing. This may have led to a sharp increase in the number of people being tested and diagnosed in 2010 and 2011 (i.e., the two pre-intervention phases). The later stages of the HIV testing promotion campaign in 2012–2013 coincided with the intervention phase. We suspect that, by this time, there may have been a lower proportion of undiagnosed HIV cases, which led to fewer newly diagnosed individuals in the intervention phase compared to the pre-intervention phase. Compared to the post-intervention 2012 phase (*n* = 215), the post-intervention 2013 phase experienced an additional but modest decrease in the number of new HIV cases (*n* = 199).

The concomitant testing promotion campaign in Guangxi may have potentially uncovered the sickest individuals first, who would have been a part of the pre-intervention outcomes, and gradually identified healthier patients, who went into the post-intervention phases. This could have contributed to the observed reduction in mortality in our study. However, the fact that the observed mortality went from above 35% before to below 13% after the implementation of the simplified test and treat intervention among the subset of patients with CD4 count ≤ 350 cells/mm^3^ or missing CD4 but reported as AIDS cases is reassuring in this regard.

We noticed that from the pre-intervention 2010 phase to the pre-intervention 2011 phase the proportion of patients initiating ART within 30 d of HIV confirmation increased from 9% to 20.1% and that overall ART initiation increased from 27% to 48.7%; in other words, ART initiation doubled, but mortality remained unchanged from the pre-intervention 2010 phase to the pre-intervention 2011 phase. This might be explained if the pre-intervention increase in ART initiation comprised mainly healthier patients, while the sickest patients continued to die at the same rate. The simplified test and treat intervention is likely to speed up treatment initiation effectively but may also change who gets treated. Please also note that though ART initiation doubled from the first to the second pre-intervention phase, ART coverage was still very low and therefore not sufficient to favorably impact mortality. The improved ART coverage from the first to the second pre-intervention phase should not undermine the effect of the intervention—although it may change in part the “mechanism” of the effect and the nature of the intervention. The fact that ART coverage within 30 d of HIV diagnosis increased sharply to 81.4% in the post-intervention 2012 phase and remained at 80% in the post-intervention 2013 phase is unlikely to be due solely to time secular trends; rather, we believe that the intervention was a major contributor to the increase in ART coverage.

In 2013, WHO changed its ART guidelines to recommend ART initiation for patients with CD4 count ≤ 500 cells/mm^3^, rather than at the previously recommended threshold of 350 cells/mm^3^ [33]. It is estimated that these revised guidelines have increased the total number of ART-eligible people in low- and middle-income countries from 16.7 million to 25.8 million [34]. In resource-limited settings, using CD4 testing as the basis for HIV staging increases the demands on clinic and laboratory staff compared to clinical staging. However, some jurisdictions in China have already embraced universal ART eligibility, irrespective of CD4 cell count, as proposed in our study [35]. Such a policy maximizes the benefits of ART in reducing HIV disease progression to AIDS and premature death and at the same time reduces HIV transmission and thereby maximizes the potential of the treatment as prevention strategy [36–39].

The HIV epidemic in China presents unique challenges. Because of the country’s size, a low national prevalence of <1% still translates into a very large HIV-positive population of approximately 780,000 [40]. In Guangxi, the epidemic is characterized by a high proportion of late-diagnosed cases, and delayed ART initiation due to the additional time required for progressing through the sequential steps of the cascade of care. With a simplified cascade, both diagnosis and ART initiation occurred earlier, resulting in enhanced engagement in ART and decreased mortality. We designed the simplified test and treat intervention specifically for the context of China’s HIV epidemic and health care structure, and, as a result, the specifics of the intervention procedures and results may not be fully generalizable to other countries. Nevertheless, the intervention targets the issues of late diagnosis and procedural barriers that delay care, which are common challenges in HIV programs worldwide. We believe that our experience is beneficial to the global community in that it provides evidence in support of the feasibility and clinical benefits of the test and treat strategy, particularly for settings where late diagnosis and treatment are common.

Although the pre-intervention/post-intervention study design allowed for control of some hospital-based characteristics, assumptions about the simplified test and treat intervention’s causal effects must be treated with caution. Internal validity is affected by history, maturation, and Hawthorne threats. Implementing a package of interventions also prevents the evaluation of the relative contribution of each of the individual components on the overall observed effect. Late diagnosis (CD4 count ≤ 200 cells/mm^3^) of HIV is a key barrier to reducing mortality and morbidity, which we could not evaluate fully in this study. In the intervention phase, we used data collected over the course of standardized care, while outcomes in the two pre-intervention phases were assessed retrospectively. The pre-intervention data monitoring was less thorough than the data monitoring in the two post- intervention phases, which may have had an impact on data quality. During the two pre-intervention phases, reporting of outcomes such as mortality may have been delayed, resulting in a lower reported baseline mortality and an underestimation of the impact of the intervention.

China has begun expanding the simplified test and treat intervention to 12 additional counties in nine provinces. Further evaluation of the feasibility and acceptability of this intervention will provide additional evidence for national and international policymakers. Early diagnosis in conjunction with prompt linkage to care could be a game changer for the HIV epidemic in China. Our results demonstrate that a simplified HIV testing approach combined with expanded access to ART, irrespective of CD4 count, can lead to a substantial reduction in mortality. Our findings support increased integration of HIV testing and treatment to optimize the potential individual and public health benefits of ART.

## Supporting Information

S1 Checklist STROBE checklist. (DOC)

S1 Text Clinical trial registration receipt. (PDF)

S2 Text Study proposal. (PDF)

S3 Text Institutional review board approval letter. (PDF)

## Abbreviations

aHR: adjusted hazard ratio
ART: antiretroviral therapy
CDC: Center for Disease Control and Prevention
China CDC: Chinese Center for Disease Control and Prevention
CI: confidence interval
CRIMS: Comprehensive Response Information Management System
HR: hazard ratio
IQR: interquartile range
LTFU: loss to follow-up
NCAIDS: National Center for AIDS/STD Control and Prevention
NFATP: National Free Antiretroviral Treatment Program
WB: Western blot
