# Supplementary material for: Simplified HIV Testing and Treatment in China: Analysis of Mortality Rates Before and After a Structural Intervention
Source: PLoS Med. 2015 Sep 8;12(9):e1001874. doi: 10.1371/journal.pmed.1001874 (PMC4562716; doi:10.1371/journal.pmed.1001874)
Supplement: S1 Checklist — (DOC) [file pmed.1001874.s001.doc]

**STROBE Guidelines Checklist**

**PMEDICINE-D-14-03887R4**

*“***Simplified HIV testing and treatment to reduce mortality in China: a structural intervention***”*

Corresponding Author:

Zunyou Wu, National Center for AIDS/STD Control and Prevention, China CDC, Beijing, China.

Tel: +86-10-58900901; Fax: +86-10-58900900; E-mail: [wuzy@263.net](mailto:wuzy@263.net) or [wuzunyou@chinaaids.cn](mailto:wuzunyou@chinaaids.cn).

| **Item**  **Description** | **Item No** |  | **Recommendation** |  | **Section** |
| --- | --- | --- | --- | --- | --- |
| **Title and abstract** | 1 |  | (*a*) Indicate the study’s design with a commonly used term in the title or the abstract |  | Title |
|  |  |  | (*b*) Provide in the abstract an informative and balanced summary of what was done and what was found |  | Abstract |
| **Introduction** |  |  |  |  |  |
| Background/  rationale | 2 |  | Explain the scientific background and rationale for the investigation being reported |  | Introduction |
| Objectives | 3 |  | State specific objectives, including any prespecified hypotheses |  | Introduction |
| **Methods** |  |  |  |  |  |
| Study design | 4 |  | Present key elements of study design early in the paper |  | Methods—Study design and participant eligibility |
| Setting | 5 |  | Describe the setting, locations, and relevant dates, including periods of recruitment, exposure, follow-up, and data collection |  | Methods—Study design, study site, study subject, Standard-of-Care (Pre-intervention) Procedures, Simplified Test and Treat Intervention Procedures,  Data Management. |
| Participants | 6 |  | (*a*) *Cohort study*—Give the eligibility criteria, and the sources and methods of selection of participants. Describe methods of follow-up |  | Methods- study subject, Standard-of-Care (Pre-intervention) Procedures, Simplified Test and Treat Intervention Procedures. |
|  |  |  | (*b*) *Cohort study*—For matched studies, give matching criteria and number of exposed and unexposed |  | N/A |
| Variables | 7 |  | Clearly define all outcomes, exposures, predictors, potential confounders, and effect modifiers. Give diagnostic criteria, if applicable |  | Methods—Data Management, Statistical analysis |
| Data sources/ measurement | 8 |  | For each variable of interest, give sources of data and details of methods of assessment (measurement). Describe comparability of assessment methods if there is more than one group. |  | Methods—Data Management, Statistical analysis |
| Bias | 9 |  | Describe any efforts to address potential sources of bias |  | Discussion—Limitations |
| Study size | 10 |  | Explain how the study size was arrived at |  | Methods—study site |
| Quantitative variables | 11 |  | Explain how quantitative variables were handled in the analyses. If applicable, describe which groupings were chosen and why |  | Methods—Statistical analysis |
| Statistical methods | 12 |  | (*a*) Describe all statistical methods, including those used to control for confounding |  | Methods—Statistical analysis |
|  |  |  | (*b*) Describe any methods used to examine subgroups and interactions |  | Methods—Statistical analysis |
|  |  |  | (*c*) Explain how missing data were addressed |  | Methods—Statistical analysis |
|  |  |  | (*d*) *Cohort study*—If applicable, explain how loss to follow-up was addressed |  | Methods—Statistical analysis |
|  |  |  | (*e*) Describe any sensitivity analyses |  | Methods—Statistical analysis |
| **Results** |  |  |  |  |  |
| Participants | 13 |  | (a) Report numbers of individuals at each stage of study—eg numbers potentially eligible, examined for eligibility, confirmed eligible, included in the study, completing follow-up, and analysed |  | Results—Study population characteristics |
|  |  |  | (b) Give reasons for non-participation at each stage |  | N/A |
|  |  |  | (c) Consider use of a flow diagram |  | Results – figure 2 |
| Descriptive data | 14 |  | (a) Give characteristics of study participants (eg demographic, clinical, social) and information on exposures and potential confounders |  | Results—Study population characteristics  Tables 1 |
|  |  |  | (b) Indicate number of participants with missing data for each variable of interest |  | Tables 1 |
|  |  |  | (c) *Cohort study*—Summarise follow-up time (eg, average and total amount) |  | Results, paragraph 1, fig 4 &5 |
| Outcome data | 15 |  | *Cohort study*—Report numbers of outcome events or summary measures over time |  | Results, table 1, fig 4 &5 |
| Main results | 16 |  | (*a*) Give unadjusted estimates and, if applicable, confounder-adjusted estimates and their precision (eg, 95% confidence interval). Make clear which confounders were adjusted for and why they were included |  | Methods—Statistical analysis, Results—Tables 2 & 3 |
|  |  |  | (*b*) Report category boundaries when continuous variables were categorized |  | Tables 1, 2 , 3 & 4 |
|  |  |  | (*c*) If relevant, consider translating estimates of relative risk into absolute risk for a meaningful time period |  | N/A |
| Other analyses | 17 |  | Report other analyses done—eg analyses of subgroups and interactions, and sensitivity analyses |  | Results—Table 1, 2, 3, 4 |
| **Discussion** |  |  |  |  |  |
| Key results | 18 |  | Summarise key results with reference to study objectives |  | Discussion |
| Limitations | 19 |  | Discuss limitations of the study, taking into account sources of potential bias or imprecision. Discuss both direction and magnitude of any potential bias |  | Discussion—Limitations |
| Interpretation | 20 |  | Give a cautious overall interpretation of results considering objectives, limitations, multiplicity of analyses, results from similar studies, and other relevant evidence |  | Discussion—Conclusions |
| Generalisability | 21 |  | Discuss the generalisability (external validity) of the study results |  | Discussion |
| **Other information** |  |  |  |  |  |
| Funding | 22 |  | Give the source of funding and the role of the funders for the present study and, if applicable, for the original study on which the present article is based |  | Acknowledgements |
