# Supplementary material for: Simplified HIV Testing and Treatment in China: Analysis of Mortality Rates Before and After a Structural Intervention
Source: PLoS Med. 2015 Sep 8;12(9):e1001874. doi: 10.1371/journal.pmed.1001874 (PMC4562716; doi:10.1371/journal.pmed.1001874)
Supplement: S1 Text — (PDF) [file pmed.1001874.s002.pdf]

Protocol Registration Receipt
06/25/2013

Pilot Study of Immediate HIV Treatment in Guangxi, China

This study is currently recruiting participants.
Verified by National Center for AIDS/STD Control and Prevention, China CDC, May 2013

Sponsor:	National Center for AIDS/STD Control and Prevention, China CDC
Collaborators:	Guangxi Center for Disease Control and Prevention<br>AbbVie
Information provided by (Responsible Party):	National Center for AIDS/STD Control and Prevention, China CDC
ClinicalTrials.gov Identifier:	

Purpose

The aim of the study is to measure the effectiveness of a pilot program in Guangxi, China to decrease mortality related to HIV/AIDS.

The study's proposed mechanism of decreasing mortality rates is to shorten the time between initial HIV screening and ART implementation to within two weeks.

The study population consists of participants who received an initial HIV infection diagnosis within the study period. Medical institutions will provide "one-stop services" by following detailed guidelines regarding reporting of positive HIV antibody screenings, further testing procedures, and treatment referrals in accordance with a pre-determined timetable. In addition, additional strategies focusing on policy development, medical personnel training, and a broad general public education campaign will be implemented.

Main assessment measures are HIV/AIDS-related mortality rates, treatment coverage, and health outcomes.

Condition	Intervention	Phase
HIV	Behavioral: Immediate post-screening	N/A

Condition	Intervention	Phase
Acquired Immunodeficiency Syndrome	treatment education	

Study Type: Interventional
Study Design: Health Services Research, Single Group Assignment, Open Label, N/A, Efficacy Study
Official Title: Pilot Study of Immediate HIV Treatment by Means of "One-stop Service" in Hospital in Guangxi, China

Further study details as provided by National Center for AIDS/STD Control and Prevention, China CDC:

Primary Outcome Measure:

- mortality and treatment coverage [Time Frame: 12 months] [Designated as safety issue: Yes]

Secondary Outcome Measures:

- mortality and treatment coverage [Time Frame: 36 months] [Designated as safety issue: Yes]

Estimated Enrollment: 1000
Study Start Date: July 2012
Estimated Study Completion Date: July 2015
Estimated Primary Completion Date: July 2013

Arms	Assigned Interventions
Experimental: Two counties: Zhongshan and Pubei<br>Immediate post-screening treatment education for HIV-positive participants residing in the Zhongshan and Pubei pilot sites in the "Treat-All" HIV Pilot Program	Behavioral: Immediate post-screening treatment education<br>immediate treatment education after screening to decrease time from initial HIV screening to treatment implementation

The aim of the study is to measure the effectiveness of a pilot program in Guangxi, China to decrease mortality related to HIV/AIDS. In past years, Guangxi has experienced a relatively high rate of late HIV diagnoses, which has contributed to a significant proportion of HIV/AIDS-related deaths occurring in the same year of initial diagnosis. The study's proposed mechanism of decreasing mortality rates is to shorten the time between initial HIV screening and ART implementation to within two weeks.

Two pilot sites were selected based on past core assessment indicators. The study population consists of participants who received an initial HIV infection diagnosis within the study period. Medical institutions will provide "one-stop services" by following detailed guidelines regarding reporting of positive HIV antibody screenings, further testing procedures, and treatment referrals in accordance with a pre-determined timetable. In addition, additional strategies focusing on policy development, medical personnel training, and a broad general public education campaign will be implemented.

The study will be performed from July,2012 to July,2015. Main assessment measures are HIV/AIDS-related

mortality rates, treatment coverage, and health outcomes.

Eligibility

Genders Eligible for Study: Both

Inclusion Criteria:

- New diagnosis of HIV infection as defined by having positive HIV antibody screening results between July 1, 2012 and July 1, 2015 OR
- Having a current residential address inside of pilot site limits

Exclusion criteria:

- Current residing outside of the borders of the designated study sites

Contacts and Locations

Contacts

Zunyou Wu, PhD   +86-10-5890-0901   wuzunyou@chinaaids.cn

Locations

China

Zhongshan Center for Disease Control   Recruiting
Zhongshan, China

China, Guangxi

Pubei Center for Disease Control   Recruiting
Pubei, Guangxi, China

Investigators

Study Chair: Zunyou Wu, PhD   National Center for AIDS/STD Control and Prevention

More Information

Responsible Party: National Center for AIDS/STD Control and Prevention, China CDC
Study ID Numbers: Treat-All HIV Pilot
Health Authority: China: Ministry of Health
