# Supplementary material for: Simplified HIV Testing and Treatment in China: Analysis of Mortality Rates Before and After a Structural Intervention
Source: PLoS Med. 2015 Sep 8;12(9):e1001874. doi: 10.1371/journal.pmed.1001874 (PMC4562716; doi:10.1371/journal.pmed.1001874)
Supplement: S2 Text — (PDF) [file pmed.1001874.s003.pdf]

PI: Zunyou Wu, MD, PhD
National Center for AIDS/STD Control & Prevention
Chinese Center for Disease Control and Prevention

Pilot Study of Expedited HIV Treatment in Guangxi, China（June 2012)

I. Background

In light of the severity of Guangxi's HIV epidemic, this study proposes a new intervention to expedite and increase linkage to HIV care with the aim of reducing HIV/AIDS-related mortality. The national and provincial Centers for Disease Control and Prevention (CDC) are committed to reducing deaths by AIDS in Guangxi. In recent years, national policies have been enacted to reinforce HIV prevention efforts, including the 12th Five-Year Action Plan: China's Proposal to Limit and Prevent AIDS.[1] In 2011, the province of Guangxi reported 3,852 AIDS-related deaths, the highest number among all provinces in China and 22% of the national total.[2] To address this problem, the proposed structural intervention will streamline the delivery of testing procedures for newly-identified HIV-positive patients.

The benefits of antiretroviral therapy (ART) for treating HIV-positive patients in developing countries has been extensively reported in the literature.[3-6] However, in practice, patients are lost at each step along the continuum of HIV care.[7] In Guangxi, only 85% of those who have a positive HIV confirmation test result are notified of their HIV test results. Furthermore, only 62% of individuals who are notified of their HIV diagnosis receive CD4 testing. Since CD4 levels are used to determine ART eligibility, we estimate that nearly 80% of newly-identified, ART-eligible patients in Guangxi are not initiated on ART.[8] Throughout the HIV care continuum, patients experience significant delays in receiving confirmation and CD4 testing. According to the current field research, the timeframe from a positive HIV point-of-care (POC) screening to confirmation of a new HIV case was an average of 1-2 weeks, extending up to 3 weeks. Delays in confirming a positive HIV diagnosis prevent timely implementation of ART.

Furthermore, these missed opportunities for patient engagement in HIV care ultimately translate into high mortality rates.Early loss to follow-up along the continuum of HIV care is reflected in late AIDS diagnoses and high levels of preventable mortality. From 2007-2011, Guangxi's late diagnoses (CD4 $\leq$200) accounted for a high percentage of all diagnoses: 28.9%, 26.1%, 33.7%, 36.3%, 35.7%, respectively.[2] The median time to death after initiating ART is 5 months, and 79% of those who die of AIDS had never received ART.[8] With less than 1 in 3 treatment-eligible patients in Guangxi receiving ART, AIDS mortality is unacceptably high.

Improving HIV treatment outcomes requires both patient and provider motivation and commitment. A challenge that must be addressed is poor provider motivation to engage and retain HIV-positive patients in care, resulting in inadequate communication between providers and patients regarding treatment needs and risk management. Previous studies have shown that financial incentives can help improve provider motivation.[10] Monetary incentives for providers can potentially to achieve increased recruitment into treatment, higher rates of retention, and

greater treatment success at moderate programmatic costs. As such, the evaluation of incentives for providers is important and will provide evidence relevant to policy decisions globally.

Current Standard of Care

Under the existing protocol, a patient for whom an HIV test has been ordered will first be screened using the ELISA or rapid test. If a patient has a positive rapid test result and is available for further testing, a second blood specimen is collected and sent to the local city-level CDC for Western blot confirmatory testing. The average turnaround time for a confirmation test is 10-15 days after the blood sample collection.

Providers collect basic patient contact information and report all confirmed HIV-positive cases through China's national infectious disease case reporting system (covering all notifiable diseases including HIV infection and AIDS40). CD4 tests are usually performed at a subsequent, independent visit to the City CDC using a BD FACSCountTM flow cytometer (Becton Dickenson, USA). The estimate time to a CD4 test result is 10-15 days. When CD4 test results are available, patients are located again to be notified of the CD4 test results and to be evaluated for ART eligibility. The current national CD4 threshold for ART eligibility in China is CD4 ≤350. Patients who are not eligible for ART (CD4 > 350) are followed every 6 months to monitor changes in treatment eligibility. Follow-up visits involve a blood draw for a CD4 test that is conducted at the City CDC.

Patients who are eligible for ART (CD4 ≤ 350) are encouraged to seek HIV care at designated treatment facilities part of China's national ART program. Prior to initiating ART, patients are required to complete a physical health exam, tests of liver and kidney function, screening for co-morbidities, and a series of counseling sessions intended to educate patients on the importance of ART adherence and transmission prevention. After ART is initiated, patients are followed every 3 months to monitor drug side effects and ART adherence. CD4 levelsare re-tested every 6 months. After initiating ART, patients are offered HIV viral load testing at the City CDC once a year. Numeration of viral DNA/RNA copies is performed using a COBAS®AmpliPrep /COBAS®TaqMan® HIV-1 test (v2.0, Roche Molecular Systems, Inc., USA).

II. Site and Participant Selection

Site Selection

Two sites (Zhongshan county and Pubei county) have been selected for this pilot study. In previous years, these two sites reported high numbers of estimated deaths following a same-year diagnosis.

Participant Selection

The initial participant enrollment period will be from July1, 2012 to June 31, 2013.Participants will meet at least one of the following two eligibility criteria:

1. Received two positive HIV antibody screening results, including a quick blood test or enzyme-linked immunosorbent assay screening examinations in line with the National HIV testing technical specifications (2009 Revision) measurement methods during the study period.

2. Received a previously confirmed diagnosis of HIV seropositivity and had not initiated ART.

Exclusion criteria:

1. Current residing outside of the borders of the designated study sites.

Study design

The study will follow a self-controlled cohort design. The intervention phase will be compared with the existing SOC at the two sites.

III. Intervention

a. sites

The pre-intervention control phase will follow the SOC procedures as described above.

General framework for the 2 sites in intervention phase :

1. Upon a positive HIV screening result, the discovering medical institution will provide an initial counseling session with the patient.
2. The discovering medical institution will refer the patient to the county hospital to begin ART eligibility screening and treatment following informed consent, adherence education, routing testing, and other appropriate medical procedures.
3. Each Wednesday, the county hospital will provide physician consultations regarding ART, blood sample collection for confirmation and CD4 cell count testing, spouse or partner screening, pre-treatment physical examinations, treatment of opportunistic infections, and other related services.
4. Each Wednesday, the county-level CDC will deliver blood samples to the city-level CDC for same-day laboratory testing.
5. At the following Friday, the city-level CDC will deliver a feedback report to the county-level CDC. The county-level CDC will send results to county hospitals to begin appropriate ART regimens.

b. Public Education

The following strategies will be used to promote the message of "more detection and early treatment" among the public. Multiple types of media channels and avenues will be used including:

a. Developing, producing, and printing early detection and early treatment educational materials, including posters and small brochures.
b. Setting up billboards near all medical and health institutions to instruct on the benefits of early detection and early treatment.
c. Directly consulting and educatingpatients who were previously diagnosed with HIV and have not initiated treatment.

c. Incentive Mechanism

A key to the effectiveness of the intervention is high treatment referral rates. An incentive system will be implemented to increase motivation among the medical staff and to improve the success rate of referrals as well as to explore the effectiveness of incentivizing junior health care providers. The study has set two incentive mechanisms for providers at the provincial, city, and county levels. A provider who identifies a screen-positive patient and refers him/her a county hospital will receive 50 CNY when the patient begins ART. ART providers will receive 30 CNY per ART initiation.

d. Training

National, provincial, and county staff directly involved in the project implementation will participate in program training and specialized training. All medical personnel at institutions within the two pilot site limits will receive training on HIV/AIDS policy and basic knowledge within the first two months of the study. The training is the responsibility of the county ministry of health. In addition, additional strategies for policy development and a broad general public education campaign will be developed and implemented.

IV. Outcome measures

Data will be collected and stored according to existing procedures. The two online unified databases are the HIV/AIDS case reporting database and the National Free Antiretroviral Treatment database. The two major assessment indicators are:

1) Mortality of participants who are newly diagnosed during the study period

Numerator: Number of all-cause deaths during the study period
Denominator: Number of HIV/AIDS patients followed during the study period

2) Proportion of newly diagnosed HIV-positive participants who receive ART

Numerator: Number of patients who are diagnosed with HIV and initiate ART during the study period
Denominator: Number of newly reported HIV-positive participants during the study period

The analysis was conducted at the end of 12 months and 36 months after the initiation of the intervention.

V. Regulatory Requirements and Informed Consent

The study has been reviewed by the Institutional Review Board of the National Center for AIDS/STD Prevention and Control, China CDC.The trial will be conducted in compliance with protocol, International Conference of Harmonization (ICH) guidelines for Good Clinical Practice (GCP), and applicable international, federal, state, and local regulatory requirements.

Prior to enrollment, study procedures and terms of participation will be explained to each participant in a private location. The information will be provided in the local dialect at an appropriate language level. Participants will have designated opportunities to ask questions about the study and the consent form. Participants will be informed that they are free to withdraw from the study at any time without penalty or prejudice. Voluntary participants will sign the informed consent form. The hospital or research staff member administering the informed consent will also sign his or her name. After signing the consent form, participants will be provided with a copy of the signed and dated form for their records. This process will be documented in the participant’s study file.

VI. Organization and Management

This proposed study will be implemented by the Guangxi Center for Disease Control and Prevention, Guangxi Ministry of Health, the appropriate county Centers for Disease Control, and the study site hospitals. Leadership, coordinator, and expert technical consultation will be provided by the National Center for AIDS/STD Prevention and Control of the Chinese CDC. To ensure study fidelity, a leadership and management team will be formed comprising the following research and provider staff members.

Leadership and Coordination Team:

Leader: Xinhua Sun
Deputy Leader: Wenkui Geng
Members: Gang Xia, Yahui Jiao, XianminGe, Chi Chen, Zunyou Wu, Zhenzhu Tang, Weiguang Tang, JinZheng, XuejunQiu, ShengLuo

Technology and Implementation Team

Leader: Zunyou Wu
Deputy Leaders: Zhenzhu Tang, Xiaojun Deng, Fengyao Wu
Members: Zhongfu Liu, Jiangping Sun, Yurong Mao, Yan Cui, Keming Rou, Yan Zhao, Can Rui Jin, Wei Guo, Jian Li, Xia Jin, Dongqing Ye, XunZhuang, ZhihaoMeng, Ruichao Lu, Shaobiao Huang, Zhiyong Chen, Zhirong Tang, Hongman Zhang, Qiuying Zhu, Youshou Li, Xiuqing Huang, Shaohui Wu, Guide Nong
Secretaries: Yurong Mao, Yan Zhao
Technical Advisors: Xiaoqiu Qi, Hong Shang, Ruotao Wang

VII. Schedule of Research Events

May-June 2012: Discussion and finalization of the study intervention
June 2012: Site preparation and personnel training
July 2012: Opening of the study enrollment period.
June2013: Collection of data for the interim analysis.
September-October 2013: Preparation 1-year interim analysis and report.

Bibliography:

1. General Office of the State Council. China's 12th Five-Year Action Plan for the Containment and Control of HIV/AIDS. Beijing: General Office of the State Council; 2012.
2. National Center for AIDS/STD Control and Prevention, China CDC. 2011 annual report of national AIDS/STD statistics on epidemics and program implementation. Beijing: National Center for AIDS/STD Control and Prevention, China CDC; 2012.
3. Gonzalez MA, Martin L, Munoz S, Jacobson JO. Patterns, trends and sex differences in HIV/AIDS reported mortality in Latin American countries: 1996-2007. BMC Public Health. 2011; **11**: 605.
4. Mills EJ, Bakanda C, Birungi J, Chan K, Ford N, Cooper CL, et al. Life expectancy of persons receiving combination antiretroviral therapy in low-income countries: a cohort analysis from Uganda. Ann Intern Med. 2011; **155**(4): 209-16.
5. Tassie JM, Malateste K, Pujades-Rodriguez M, Poulet E, Bennett D, Harries A, et al. Evaluation of three sampling methods to monitor outcomes of antiretroviral treatment programmes in low- and middle-income countries. PLoS One. 2010; **5**(11): e13899.
6. Nolan S, Milloy MJ, Zhang R, Kerr T, Hogg R, Montaner J, et al. Adherence and plasma HIV RNA response to antiretroviral therapy among HIV-seropositive injection drug users in a Canadian setting. AIDS Care. 2011; **23**(aa42b4cb-d5d6-d86e-5293-f81dd3c70f97): 980-7.
7. Nash D, Wu Y, Elul B, Hoos D, El Sadr W. Program-level and contextual-level determinants of low-median CD4+ cell count in cohorts of persons initiating ART in eight sub-Saharan African countries. AIDS. 2011; **25**(12): 1523-33.
8. National Center for AIDS/STD Control and Prevention, China CDC. Analysis of HIV/AIDS testing and treatment program in Guangxi. Beijing: National Center for AIDS/STD Control and Prevention, China CDC; 2012.
9. Yip WC, Hsiao W, Meng Q, Chen W, Sun X. Realignment of incentives for health-care providers in China. Lancet. 2010; **375**(9720): 1120-30.
10. Wu Z. Training of grass-root health care providers and family cares for providing care to HIV infected in rural China. Beijing: National Center for AIDS/STD Control and Prevention, China CDC; 2005.
