# Supplementary material for: Simplified HIV Testing and Treatment in China: Analysis of Mortality Rates Before and After a Structural Intervention
Source: PLoS Med. 2015 Sep 8;12(9):e1001874. doi: 10.1371/journal.pmed.1001874 (PMC4562716; doi:10.1371/journal.pmed.1001874)
Supplement: S3 Text — (PDF) [file pmed.1001874.s004.pdf]

中国疾病预防控制中心性病艾滋病预防控制中心
NATIONAL CENTER FOR AIDS/STD CONTROL AND PREVENTION, CHINESE CENTER FOR DISEASE CONTROL AND PREVENTION

155 Changbai Road, Changping District
Beijing 102206, P. R. China
Website: www.Chinaaids.cn

中国疾病预防控制中心性病艾滋病预防控制中心

伦理委员会英文批件

（INSTITUTION REVIEW BOARD APPROVE LETTER）

Project No: X120717220

PROJECT TITLE: A pilot of Treat-all strategy for reducing the mortality among HIV infected patients in Guangxi

PRINCIPAL INVESTIGATOR: Zunyou Wu

INSTITUTE: National Center for AIDS/STD Control and Prevention, Chinese Center for Disease Control and Prevention, P. R. China

FUNDING AGENCY: Abbvie China

DATE FOR WHICH REVIEWED: July 17, 2012

DATE APPROVED: August 7, 2012

The INSTITUTIONAL REVIEW BOARD of NATIONAL CENTER FOR AIDS/STD CONTROL AND PREVENTION, CHINESE CENTER FOR DISEASE CONTROL AND PREVENTION, has reviewed the proposed use of human subjects in the above-mentioned project. The right and the welfare of the subject are adequately protected; the potential risks are outweighed by potential benefits.

Our IRB, National Center for AIDS/STD Control and Prevention, Chinese Center for Disease Control and Prevention (China CDC) is registered with the U.S. Office for Human Research Protections, IRB00002276, and has a Federal wide Assurance. FWA00002958.

Signature: Ruotao Wang

Ruotao Wang

Chair, Institutional Review Board of NCAIDS, CCDC

Date: August 7, 2012
